# A *5*+*1* assemble-to-activate mechanism of the Lon proteolytic machine

**DOI:** 10.1038/s41467-023-43035-2

**Published:** 2023-11-13

**Authors:** Shanshan Li, Kan-Yen Hsieh, Chiao-I Kuo, Tzu-Chi Lin, Szu-Hui Lee, Yi-Ru Chen, Chun-Hsiung Wang, Meng-Ru Ho, See-Yeun Ting, Kaiming Zhang, Chung-I Chang

**Affiliations:** 1 Department of Urology, The First Affiliated Hospital of USTC, MOE Key Laboratory for Cellular Dynamics, Center for Advanced Interdisciplinary Science and Biomedicine of IHM, Division of Life Sciences and Medicine, University of Science and Technology of China, 230001 Hefei, China; 2 Institute of Biological Chemistry, Academia Sinica, Taipei, 11529 Taiwan; 3 Institute of Molecular Biology, Academia Sinica, Taipei, 11529 Taiwan; 4 Institute of Biochemical Sciences, College of Life Science, National Taiwan University, Taipei, 10617 Taiwan

**Keywords:** Proteases, Cryoelectron microscopy

## Abstract

Many AAA+ (ATPases associated with diverse cellular activities) proteins function as protein or DNA remodelers by threading the substrate through the central pore of their hexameric assemblies. In this ATP-dependent translocating state, the substrate is gripped by the pore loops of the ATPase domains arranged in a universal right-handed spiral staircase organization. However, the process by which a AAA+ protein is activated to adopt this substrate-pore-loop arrangement remains unknown. We show here, using cryo-electron microscopy (cryo-EM), that the activation process of the Lon AAA+ protease may involve a pentameric assembly and a substrate-dependent incorporation of the sixth protomer to form the substrate-pore-loop contacts seen in the translocating state. Based on the structural results, we design truncated monomeric mutants that inhibit Lon activity by binding to the native pentamer and demonstrated that expressing these monomeric mutants in *Escherichia coli* cells containing functional Lon elicits specific phenotypes associated with *lon* deficiency, including the inhibition of persister cell formation. These findings uncover a substrate-dependent assembly process for the activation of a AAA+ protein and demonstrate a targeted approach to selectively inhibit its function within cells.

## Abstract (web summary)

Many AAA+ (ATPases associated with diverse cellular activities) proteins function as a hexamer to remodel protein substrates. Here, the authors report the discovery of a pentameric form of the Lon AAA+ protease and show that it plays a role in the substrate-dependent activation of the AAA+ protein.

## Introduction

AAA+ proteins constitute a large superfamily of ATPases with remodeling activities for substrates ranging from proteins and nucleic acids to their multi-component complexes. They are conserved across all kingdoms and function as molecular machines that are involved in protein degradation, protein unfolding, microtubule dynamics, DNA replication, chromatin remodeling, and ribosomal RNA processing^1–3^. A hallmark feature of the AAA+ proteins is their hexameric assembly, in which the conserved ATPase subunits are arranged in a unique close-ring structure that interacts with the substrate through a central pore^1^. The substrate is translocated, or moved across the central pore of the ring, by a process driven by coordinated structural changes of the ATPase subunits triggered by ATP binding and hydrolysis^4^. Based on the cryo-EM structures of many substrate-bound AAA+ proteins, the substrate is contacted by the pore-loop strands from 3 to 5 ATPase subunits, which always form a continuous right-handed spiral staircase arrangement around the substrate; the right-handed substrate-engaging helical complex is seamed by 1–3 ATPase subunits with non-contacting pore-loop strands to maintain a close-ring structure with mixed ATP/ADP-bound states.

The Lon protease is a AAA+ protein found in prokaryotes, archaea, and eukaryotic organelles^5^. Lon is involved in cellular protein homeostasis by degrading damaged or misfolded abnormal proteins, which prevents these unwanted protein species from forming toxic aggregates. Lon also participates in many cell regulation pathways by degrading specific regulatory proteins. The *lon* mutation in *E. coli* affects various physiological processes, resulting in several classic phenotypes. They include the mucoid colony formation, due to the accumulation of the activity of RcsA, a substrate of Lon, leading to the overproduction of capsular polysaccharide^6^. Moreover, Lon-deficient bacteria are sensitive to ultraviolet (UV) light, which results in the eponymous “long” filamentation after exposure to UV irradiation^7,8^. UV treatment induces DNA damage, leading to an increase in the expression of the cell-division inhibitor SulA^9,10^, which is regulated by the *E. coli* Lon protein (EcoLon) through proteolysis^11^. Degradation of SulA by EcoLon allows cells to resume cell division after recovering from DNA damage. Without EcoLon, SulA levels remain high, and cells continue to grow without septation, resulting in lethal filamentation. Recently, the deletion of *lon* has been shown to reduce persister formation in *E. coli*^12–15^.

**Fig. 1:**
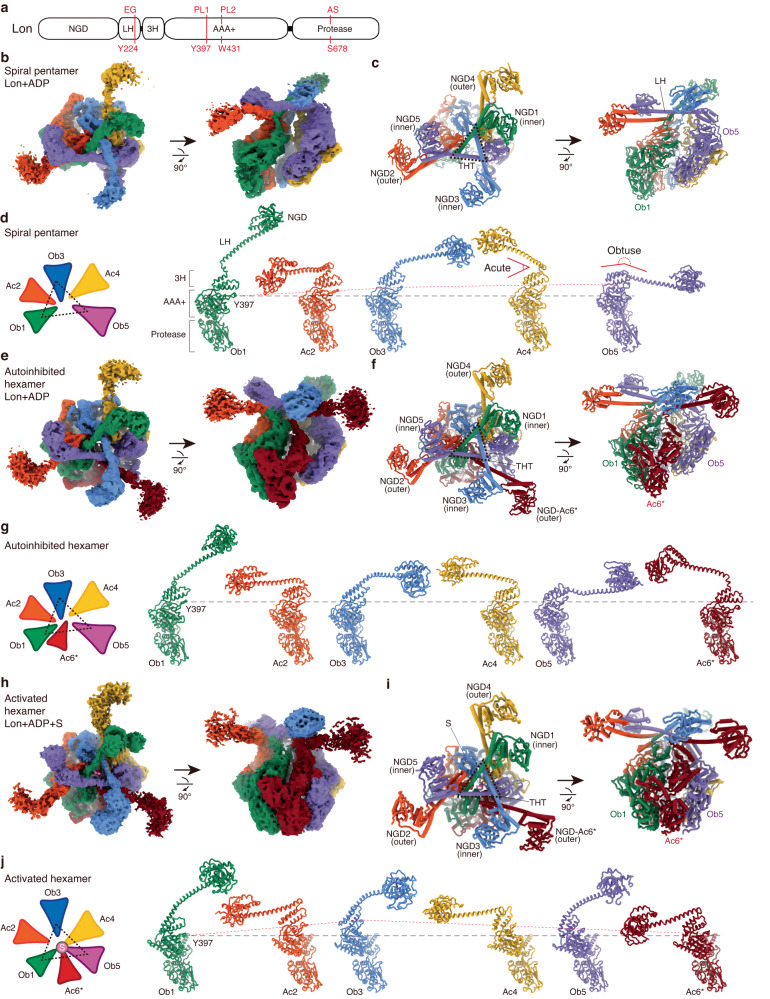
Cryo-EM structures of Lon in three assembled states. **a** Each Lon protomer consists of an N-terminal globular domain (NGD), a long helix (LH), a 3-helix (3H) bundle, a AAA+ ATPase domain, and a protease domain. The locations of the residues of the entry gate (EG), the pore loops (PL1 and PL2), and the proteolytic active site (AS) are indicated. **b**, **c** Reconstructed cryo-EM map (**b**) and overall structure (**c**) of the open-spiral pentamer of wild-type MtaLon incubated with ADP. The tensegrity helix triangle (THT) is indicated by the dashed triangle. Map contour levels in ChimeraX: NGDs of the Ob protomers, 0.1; NGDs of the Ac protomers, 0.027; LHs of the Ob protomer, 0.1; LHs of the Ac protomer, 0.05; AAA-protease domains, 0.15. **d** Arrangement diagram (left) and structures of the protomers in the pentamer, aligned based on the protease domain. The red dash lines are shown to connect the Cα atom of Y397 of Ob1 and other protomers, shown in spheres. A black horizontal line is shown for comparison. **e**, **f** Reconstructed cryo-EM map (**e**) and overall structure (**f**) of the auto-inhibited spiral hexamer in cylindrical models. Map contour levels: NGDs of the Ob protomers, 0.07; NGDs of the Ac protomers, 0.02; LHs of the Ob protomers, 0.07; LHs of the Ac protomers, 0.01; AAA-protease domains, 0.13. **g** Arrangement and structures of the protomers in the auto-inhibited hexamer. **h**, **i** Reconstructed cryo-EM map (**h**) and overall structure (**i**) of the close-ring hexamer of MtaLon-S678A:casein-ADP. Map contour levels: NGDs of the Ob protomers, 0.03; NGDs of the Ac protomers, 0.01; LHs of the Ob protomer, 0.06; LHs of the Ac protomer, 0.05; the substrate and AAA-protease domains, 0.08. **j** Arrangement and structures of the protomers in the activated hexamer. The red dashed lines are shown to connect the Cα atom of Y397 of Ob1 and other protomers.

Lon is known to form a homohexamer; each protomer is composed of multiple domains, including an N-terminal substrate-binding globular domain (NGD), a long-helix domain (LH), a three-helix bundle (3H), a AAA+ ATPase module, and a protease domain (Fig. 1a). Previous cryo-EM studies of Lon showed that their ATPase-protease regions form a close-ring hexamer with the substrate-bound ATPase modules arranged in the right-handed spiral staircase organization^16–23^. Interestingly, some of these studies also reported substrate-free structures adopting a left-handed hexameric spiral arrangement of the protomers^16,17,22,24^. It is not clear how the structural change of the AAA+ protein is induced from the substrate-free state to form the right-handed, spirally arranged substrate-pore-loop contacts.

Here, we reveal a pentameric form of Lon, identified by single-particle cryo-EM reconstructions. Our structural analysis suggests that the pentameric form is involved in the activation of Lon, which occurs during the assembly process, by interacting with the sixth monomer in a substrate-dependent manner. We are able to design two monomeric mutants that inhibit the proteolytic activity and cellular function of Lon by binding to the pentameric form, demonstrating the mechanistic role of the pentameric intermediate in the activation of the hexameric AAA+ protein.

## Results

### Identification of the Lon pentamer

In the presence of ATPγS, *Meiothermus taiwanensis* Lon (MtaLon) forms a close-ring hexameric complex with a co-purified endogenous substrate^21^. To study the structure in the substrate-free state with ATPγS, we first set out to identify MtaLon with a point mutation in the N-terminal LH domain to eliminate substrate binding while preserving the native sequence of the AAA+ and the protease domains (Fig. 1a). Using a fluorescence polarization (FP) assay, we found that MtaLon-Y224S, which carries a mutation of the entry-gate residue Tyr224 to Ser^19^, does not interact with fluorescein isothiocyanate (FITC)-labeled casein (Fig. 1a and Supplementary Fig. 1). Previously known mutation of the pore-loop-I residue, Y397A, located in the ATPase domain, also abolished substrate binding as expected; by contrast, the proteolytically deficient MtaLon-S678A mutant displayed binding to FITC-casein, with a Kd value of 1.38 ± 0.29 μM (Fig. 1a and Supplementary Fig. 1).

We conducted cryo-EM analysis on MtaLon-Y224S incubated with 5 mM of ATPγS (MtaLon-Y224S:ATPγS). To provide a comparative context, we also examined wild-type MtaLon in two states: without any nucleotide (MtaLon-Apo) and incubated with 5 mM ADP (MtaLon:ADP) (Supplementary Tables 1, 2, and 3). Unexpectedly, both MtaLon-Y224S:ATPγS and wild-type MtaLon, whether with ADP or lacking nucleotide, predominantly formed left-handed spiral pentamers or spiral hexamers, rather than closed-ring hexamers (Supplementary Figs. 2–4). Surprisingly, the pentameric form consistently emerged as the most prevalent class (Supplementary Figs. 2c, 3c, and 4c). Because the reconstructions of the MtaLon:ADP data displayed the best map quality, they are used for the following structural analysis and figure presentation of the spiral structures.

The pentameric assembly forms a left-handed open-spiral structure (Fig. 1b, c). The LH of the lowermost protomer protrudes at an obtuse angle from the first helix of the 3H subdomain of the ATPase domain (hereafter named the Ob-protomer, Ob1) (Fig. 1d). This Ob-protomer is adjoined by a protomer with the LH extending in an acute angle from the ATPase domain (the Ac-protomer, Ac2). Based on the LH orientation, this spiral complex is composed of five protomers in the “Ob1-Ac2-Ob3-Ac4-Ob5” arrangement, in a clockwise ascending direction (Fig. 1d). The open-spiral pentamer forms a large gap between the protomers Ob1 and Ob5. The pentamer forms a tensegrity helix triangle (THT), consisting of three overlapping LHs from Ob1, Ob3, and Ob5, forming an interlocked, letter A-shaped structure that tethers together five protomers (Fig. 1b, c). Owing to structural flexibility, the map densities for the five NGD domains are weak and fragmented; nevertheless, they define clearly the location of these substrate-binding domains (Fig. 1b).

### Regulated binding of the 6th protomer

The substrate-free hexameric form also adopts a left-handed spiral structure (Fig. 1e, f). It is composed of the tethered pentamer and an additional Ac-protomer, Ac6* (Fig. 1f), which binds next to the protomer Ob1 and occupies the lowermost position of the spiral (Fig. 1g). Interestingly, the 3H subdomain of Ac6* occupies the axial cavity of the spiral complex and binds to the ATPase domains of the protomers 1–5, effectively blocking the accessibility of the pore loops and forming an inactive structure (Supplementary Fig. 5).

Because the five protomers in the pentameric assembly form a tethered unit by the overlapping LHs of Ob1, Ob3, and Ob5, the hexameric Lon is likely formed by binding of the sixth protomer Ac6* to the pentameric assembly. Given that our cryo-EM analysis revealed an auto-inhibited hexameric form in the substrate-free state, could binding of the sixth protomer in the presence of substrate lead to an activated hexameric complex? To test this hypothesis, we performed single-particle cryo-EM reconstruction of the proteolytically deficient mutant MtaLon-S678A incubated with ADP and the model substrate α-casein. Indeed, 3D reconstruction of the data revealed a substrate-bound hexameric form (Supplementary Fig. 6 and Supplementary Table 4), which adopts a close-ring structure with the substrate-pore-loop contacts in a right-handed spiral-staircase-like arrangement (Fig. 1h–j and Supplementary Fig. 7).

Previously, similar close-ring hexameric structures of Lon were only reported with the presence of substrate and ATP analogs. Here, our structural analysis shows that in the substrate-bound state, the sixth protomer Ac6* binds to the pentameric assembly and bridges Ob1 and Ob5 together to form a close-ring hexamer, in which the four substrate-engaged protomers are Ac4-Ob5-Ac6*-Ob1 (Fig. 1h–j and Supplementary Fig. 7a). In this structure, Ac4, Ob5, and Ac6* engage the substrate polypeptide by the pore-loop I (Tyr397) and II (Trp431) residues, but the lowermost Ob1 contacts the substrate by pore-loop I only (Supplementary Fig. 7a, b). The three protomers Ac4, Ob5, and Ac6* are tightly packed. By contrast, the protomers Ob1, Ac2, and Ob3 are only loosely contacted by their AAA+ domains (Supplementary Fig. 7a); and their pore loops II are disordered. The rigid-body rotation pattern of the protomers 1 to 5 shares some resemblance with that in the pentameric form but not in the inactive hexameric form (Fig. 1d, g, j). All of the substrate-binding loops in the proteolytic active sites adopt an active conformation, albeit not as ordered as in the inhibitor- or substrate-bound states with ATPγS (Supplementary Fig. 7c–e)^19–21^.

**Fig. 2:**
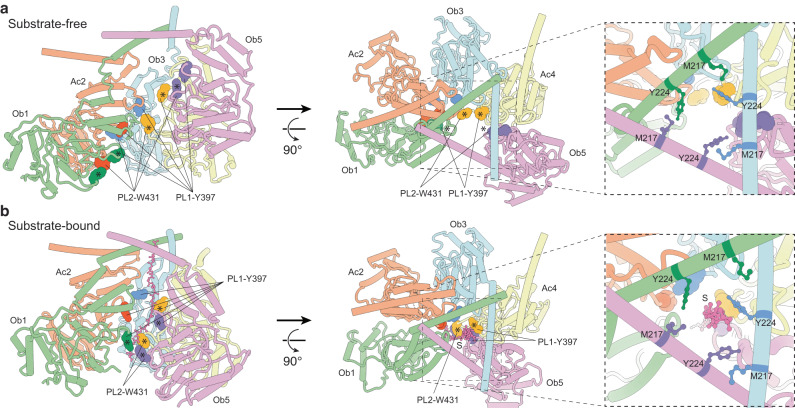
Arrangement of the pore-loop residues in the substrate-free and substrate-bound states of MtaLon. **a**, **b** Orthogonal views of the substrate-free form (**a**) and substrate-bound form (**b**) of MtaLon incubated with ADP, which show the locations of the pore-loop (PL) residues (spheres) of the protomers 1–5. The integrated protomer Ac6* in the substrate-bound form is omitted. The PL residues marked by asterisks are accessible to the entry gate in the substrate-free state. For clarity, only the region consisting of residues 208–490, which includes part of the LHs and the AAA+ domain, is shown.

In the pentameric assembly, only the pore-loop residues, Tyr397 and Trp431, of the protomers Ob1, Ac4, and Ob5 are accessible to the entry gate lined by Tyr224 and Met217 residues (Fig. 2a). Notably, they are scattered along a right-handed spiral trajectory (Supplementary Fig. 8a). In the close-ring hexameric state, the pore-loop-I Y397 residues of these protomers and Ac6* mediate substrate contacts (Fig. 2b); together they are aligned on the trajectory (Supplementary Fig. 8b). This observation suggests that Y397 may play a role during the substrate-dependent assembly process. Therefore, we performed cryo-EM analysis on the double mutant of MtaLon-Y397A/S678A and found that the mutant failed to form the substrate-bound close-ring hexamer despite the presence of α-casein and ATPγS (Supplementary Fig. 9 and Supplementary Table 5). Overall, these results support that the substrate and the pore-loop-I residues play key roles in directing the binding of the sixth protomer Ac6* to the pentamer, thereby forming a close-ring hexamer with a right-handed spiral staircase arrangement of the substrate-pore-loop contacts.

### Interconnection of three protomers

**Fig. 3:**
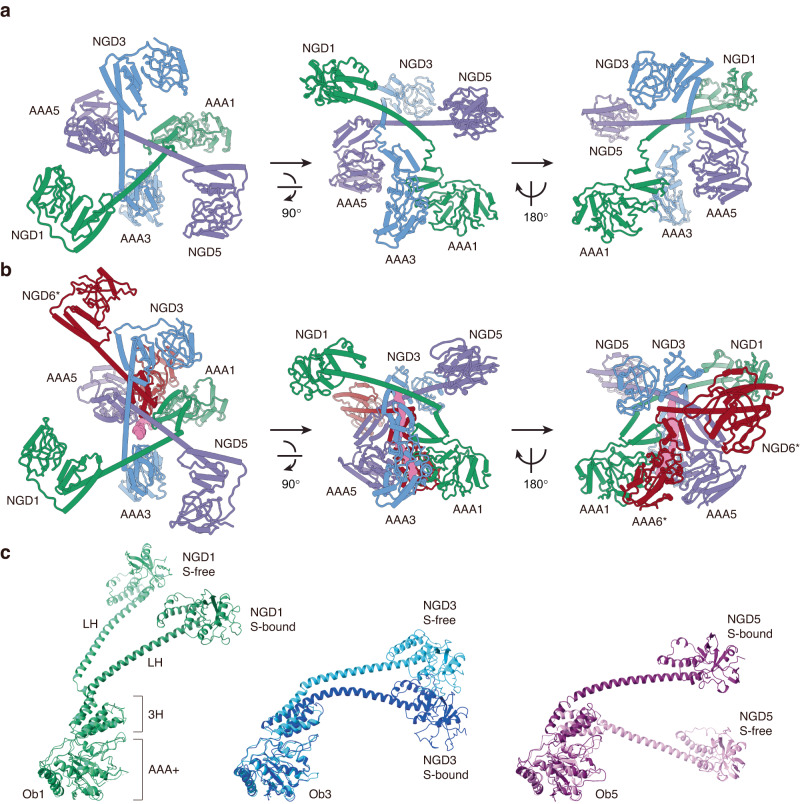
Crosslinked structures of the NGD and AAA+ domains of three Ob-protomers. **a**, **b** Structures of the overlapping long helices (LH), which connects the NGD and AAA+ domains of the protomers Ob1, Ob3, and Ob5, in the substrate-free pentamer (**a**) and in the substrate-bound close-ring hexamer (**b**), which are aligned based on the AAA3 domain. The substrate (S) is shown in surface representation and colored in magenta. The Ac2/4 protomers are omitted for clarity. **c** The protomers Ob1, Ob3, and Ob5 in the substrate (S)-free and S-bound states are superimposed based on the AAA+ domain (residues 297–490). Only residues 1–490 are shown.

The three inner NGD domains and the AAA+ domains of the protomers Ob1, Ob3, and Ob5 are non-covalently linked together by three long helices LH1, LH3, and LH5, which adopt different crosslinked THT structures in the substrate-free and substrate-bound states (Fig. 3a, b). Accordingly, structural superimposition of the open-ring pentamer and the substrate-bound close-ring hexamer based on Ob1 reveals correlated structural changes of Ob3 and Ob5 (Fig. 3c), which in turn brings about the coordinated structural changes of the Ac-protomers (Fig. 1d, j). The three crossover contacts of the THT are mediated by a conserved hydrophobic region on each LH, which consists of the quadruple L205/V209/V213/M217 residues. To test the role of the THT in the activation process, we performed cryo-EM analysis of MtaLon-M217A incubated with casein and ADP. The M217A mutation was shown to abolish substrate binding (Supplementary Fig. 1). Moreover, it was found that MtaLon-M217A:casein:ADP forms only the spiral oligomers but no close-ring hexamer (Supplementary Fig. 10 and Supplementary Table 6). These results suggest that the interconnection of the three Ob-protomers via the THT structure is required for the coordinated structural changes in the AAA+ domains, which is likely induced by the interaction of substrate protein with three inner NGDs to regulate the incorporation of the sixth protomer Ac6*.

### Monomeric mutants bound to the pentamer

**Fig. 4:**
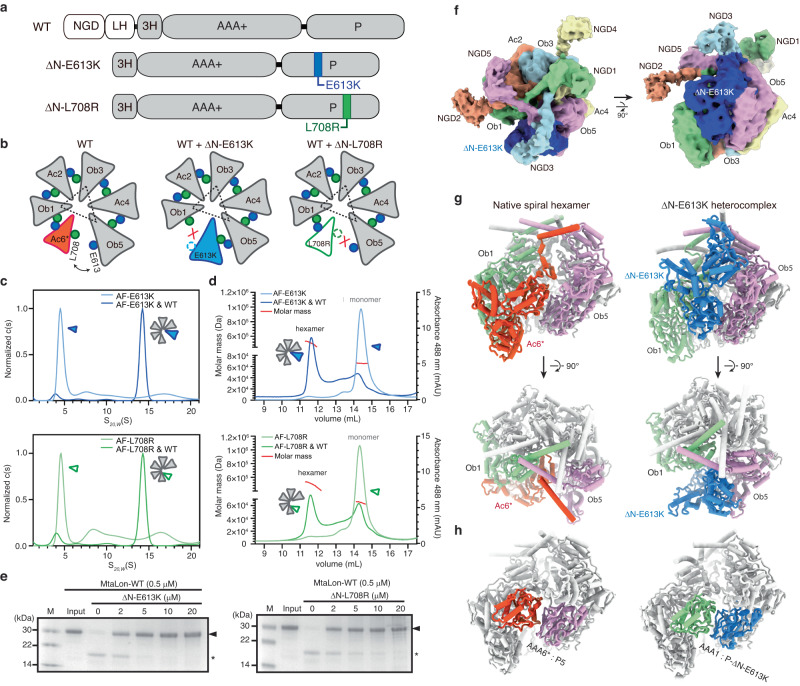
Designed monomeric mutants bind to the pentameric form and inhibit the activity of Lon. **a** Domain diagrams of the mutants ΔN-E613K and ΔN-L708R. **b** Cartoon illustrations showing the presumed binding modes of ΔN-E613K and ΔN-L708R to the pentameric MtaLon. **c** SV-AUC analysis of Alexa Fluor 488-labeled ΔN-E613K (AF-E613K), ΔN-L708R (AF-L708R), and their respective incubations with MtaLon (WT). **d** SEC-MALS analysis of the recycled SV-AUC samples. **e** Degradation of casein by MtaLon without or in the presence of the indicated amounts of ΔN-E613K and ΔN-L708R. M denotes the molecular weight marker. The “input” lane shows the substrate before the reaction. The substrate α-casein bands in the gels are indicated by the arrowheads, and the asterisks denote product fragments. The experiment was repeated three times independently with similar results. **f**, **g** Cryo-EM map (**f**) and the structure (**g**) of the heteromeric complex of ΔN-E613K bound to the native pentamer. The structure of the native spiral hexamer is also shown for comparison. Map contour levels in ChimeraX: NGDs of the Ac protomers, 0.036; NGDs of the Ac protomers and AAA-protease domains, 0.1. **h** Intermolecular interaction between the AAA+ and protease (P) domains, highlighted by the color scheme of (**g**), in the native spiral hexamer (left) and the ΔN-E613K heterocomplex (right). The NGDs are omitted from the cylindrical models for clarity. Source data are provided as a Source Data file.

If the substrate-translocating hexamer is formed by substrate-directed incorporation of the sixth protomer, an excess amount of an inactive monomeric mutant should presumably disrupt the activity and function of wild-type Lon by binding to the pentameric form and interfering with the incorporation of the sixth protomer. Therefore, we sought to create MtaLon mutants that lack the N-terminal substrate-binding domain and form monomers. We identified Glu613 and Leu708, two highly conserved residues at a hydrophobic interface between the protease domains (Supplementary Fig. 11); these residues make van der Waals contacts in all the spiral or close-ring forms. To create monomeric mutants, we designed ΔN-E613K and ΔN-L708R mutations, each of which would introduce a positive charge to the hydrophobic interface (Fig. 4a). If the ΔN-E613K and ΔN-L708R proteins are monomeric, they are expected to bind next to Ob5 and Ob1, respectively, of the open-spiral pentamer to form inactive hexamers (Fig. 4b).

We specifically labeled ΔN-E613K and ΔN-L708R mutants with Alexa Fluor 488 dye (AF488) and employed sedimentation velocity analytical ultracentrifugation (SV-AUC) and size-exclusion chromatography coupled to multi-angle light scattering (SEC-MALS) to detect the change of the hydrodynamic radius and determine the molecular mass of the peaks with absorbance signals at 488 nm, in the absence or presence of wild-type MtaLon protein (MtaLon-WT). The results of our SV-AUC and SEC-MALS experiments showed that both AF488-ΔN-E613K and AF488-ΔN-L708R mutants exist as monomers in solution (Fig. 4c, d). Upon incubation with MtaLon-WT, however, we observed a positional shift of the fluorescent peak of each mutant from monomeric to hexameric positions in the SV-AUC plots and SEC-MALS chromatograms (Fig. 4c, d). Western blot analysis of the fractions from size-exclusion chromatography also detected a similar molecular weight shift of the monomeric mutants to higher-order complexes induced by binding to MtaLon-WT (Supplementary Fig. 12). The heteromeric complexes containing the incorporated monomeric ΔN-E613K or ΔN-L708R are expected to be non-functional as they are unable to form a closed-ring structure due to the mutated residues at the binding interface (Fig. 4c). To test this hypothesis, we conducted activity assays to investigate the impact of ΔN-E613K and ΔN-L708R on the degradation of casein by MtaLon-WT in the presence of ATP. Analysis by SDS–polyacrylamide gel electrophoresis (SDS-PAGE) showed that ΔN-E613K and ΔN-L708R effectively inhibit the activity of MtaLon-WT in a dose-dependent fashion (Fig. 4e), demonstrating the inhibitory effect of these monomeric mutants in vitro.

We solved the cryo-EM structure of ΔN-E613K in complex with MtaLon-Y224S (Fig. 4f, g, Supplementary Fig. 13 and Supplementary Table 7). The structure forms a distinct spiral hexameric complex, in which ΔN-E613K binds next to Ob5 of the pentameric assembly, which is consistent with the predicted binding mode (Fig. 4b). This heteromeric hexamer distinguishes from the native spiral hexameric form, where the protomer Ac6* binds next to Ob1 (Fig. 4g). The heterocomplex also adopts an auto-inhibited spiral structure; however, unlike the native spiral hexamer, the 3H subdomain of Ob1 rather than Ac6* occupies the axial cavity of the spiral complex (Fig. 4g). In these structures, the protease domain of ΔN-E613K and the AAA+ domain of Ac6* engage heterotypic interactions with the AAA+ domain of Ob1 and the protease domain of Ob5, respectively (Fig. 4h). Altogether, these results demonstrate the binding of a monomeric mutant to the pentameric Lon, which results in a *5*+*1* heteromeric complex.

### Monomeric mutants acting as inhibitors

**Fig. 5:**
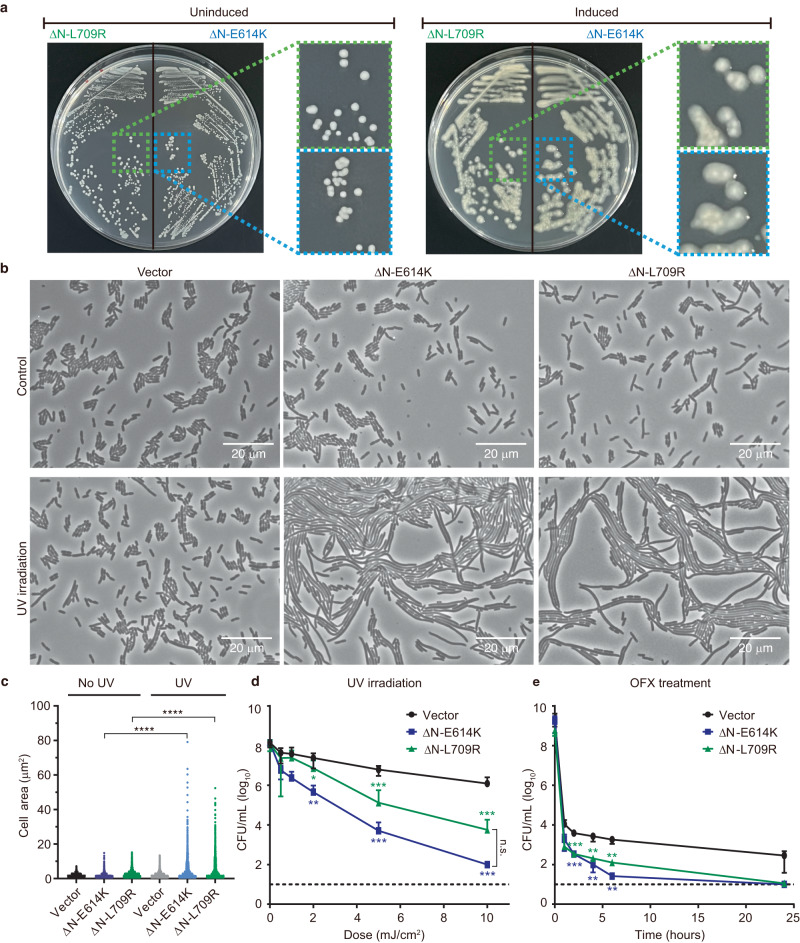
Expression of monomeric mutants in *E. coli* cells elicits a range of phenotypes associated with Lon deficiency. **a** Pictures showing the colonial morphology of *E. coli* MG1655 cells transformed with the EcoLon-ΔN-E614K or -ΔN-L709R plasmids. Cells without induction by L-arabinose appear as non-mucoid colonies when cultured on agar media (left), but cells with protein overexpression induced by L-arabinose form mucoid colonies (right). **b** Images of MG1655 cells transformed with a control vector or plasmids expressing EcoLon-ΔN-E614K or -ΔN-L709R, without or with UV treatment. **c** Calculated cell-size distributions of the transformed MG1655 cells from the UV experiments in (**b**). The quantified region contains at least 500 cells, which were detected using a minimum cell area of 0.9 µm^2^. Four asterisks (****) denote *P* < 0.0001; the *P*-values approach zero. **d** UV sensitivity of transformed MG1655 cells. Cells expressing EcoLon-ΔN-E614K or -L709R were exposed to the indicated doses of UV irradiation at 254 nm. The *P*-values for ΔN-E614K at UV doses of 2, 5, and 10 mJ are 0.0035, 0.000021, and 0.00077, respectively; the corresponding *P*-values for ΔN-L709R are 0.025, 0.000038, and 0.0008, respectively. **e** Persister levels of transformed MG1655 cells treated with ofloxacin (OFX). Cells expressing EcoLon-ΔN-E614K or -L709R were treated with 5 μg/mL OFX for 0, 1, 2, 4, 6, and 24 h. The *P*-values for ΔN-E614K at 2, 4, and 6 h are 0.000072, 0.0052, and 0.0024, respectively; the corresponding *P*-values for ΔN-L709R are 0.000073, 0.0063, and 0.0032, respectively. CFU of cells treated with UV exposure (**d**) or OFX (**e**) were det**e**rmined by plating on LB agar media with L-arabinose. The dashed line indicates the limit of detection for CFU counts. Data are presented as mean with SD (as shown by error bars) of three independent measurements. Statistical analysis was performed using one-way ANOVA with Tukey’s multiple comparisons test: *, **, and *** represent *P* < 0.1, *P* < 0.01, and *P* < 0.001, respectively; n.s. = not significant. Source data are provided as a Source Data file.

Given that the monomeric Lon mutants completely inhibit Lon activity in vitro (Fig. 4e), overexpression of these mutants in wild-type *E. coli* should phenocopy the diverse effects of the *lon* deficiency in bacteria. Therefore, we investigated the in vivo inhibitory effects of EcoLon-ΔN-E614K and -ΔN-L709R, which carry the equivalent mutations of E613K and L708R in MtaLon, respectively. We transformed *E. coli* strain MG1655 cells with plasmids containing the respective genes under the control of the *P*_*BAD*_ promoter (Supplementary Fig. 14). We examined the colonial morphology of transformed cells on agar plates without or with 0.5% L-arabinose, which induces overexpression of the mutant proteins. Cells transformed with EcoLon-ΔN-E614K or EcoLon-ΔN-L709R appeared as normal, non-mucoid colonies without the inducer; however, they formed mucoid colonies in the presence of the inducer (Fig. 5a). After treatment with UV irradiation and arabinose induction of EcoLon-ΔN-E614K or -ΔN-L709R expression, the cells were imaged under microscopy. The results showed a significant increase in cell length among those transformed with the EcoLon-ΔN-E614K or -ΔN-L709R plasmids in comparison to the empty vector (Fig. 5b, c). Cells overexpressing the monomeric Lon mutants became sensitive to UV irradiation (Fig. 5d). Finally, the persister levels of these cells following exposure to the antibiotic ofloxacin, which induces DNA damage by inhibiting DNA gyrase activity, were reduced significantly (Fig. 5e). Collectively, these results demonstrate that the monomeric Lon proteins disrupt the normal function of EcoLon-WT in cells.

## Discussion

**Fig. 6:**
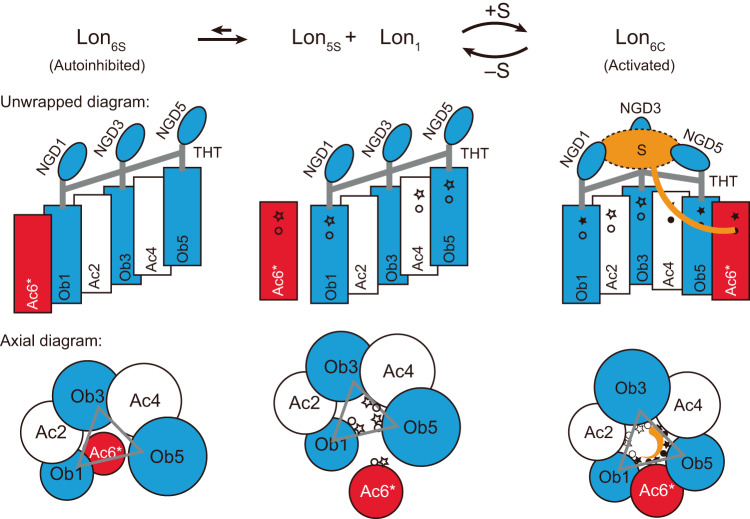
Proposed model for the substrate-dependent activation of Lon. Two diagrams depict the unwrapped and axial views of the AAA+ domains of the Lon complex in the open-spiral pentameric form (5S; center), the spiral hexameric form (6S; left), and the close-ring hexameric form (6C; right). The NGDs are shown of the Ob-protomers, colored in blue, which are linked by the tensegrity helix triangle (THT) scaffold (gray lines). The protomer Ac6* is highlighted in red. The diagrams illustrate how the binding of the substrate (S; orange) to the three NGDs and incorporation of Ac6* with Ob1 and Ob5 allosterically induce the formation of the substrate-pore-loop contacts in the universal right-handed staircase arrangement for substrate translocation. The pore-loop I and II residues accessible for substrate binding are denoted by hollow star and dot symbols, respectively. The pore-loop residues making substrate contacts are shown in solid symbols.

In this study, we report the discovery of the Lon pentamer; we confirm its functional role by designing monomeric constructs that bind to the pentamer and inhibit the Lon activity. These results suggest a substrate-dependent activation model of Lon that is mediated by a pentameric intermediate (Fig. 6). In the absence of substrate, the sixth protomer binds to the pentamer, occupying the lowest position of the spiral oligomer, resulting in the formation of an auto-inhibited spiral hexamer. However, when the substrate is present, binding of the substrate to the exposed pore loops of pentamer may induce binding of pore loops of the sixth protomer to the engaged substrate polypeptide presented by pentamer and promote rapid formation of the closed-ring hexamer with stabilized substrate interaction. Perhaps due to the transient and dynamic nature of this substrate-dependent assembly process, we did not observe any class of the substrate-bound pentameric state by our cryo-EM analyses.

Additionally, our cryo-EM structures of a series of spiral subcomplex oligomers of Lon determined by 3D reconstructions suggest a sequential pathway for the construction of the THT. In the proposed pathway, the THT is formed by sequential incorporation of the protomers, beginning with Ob1 and progressing in the clockwise direction through Ob5, and completed by the incorporation of Ac6* (Supplementary Fig. 15), whose binding mode is dependent on the substrate. The presence of a mixture of monomers and small oligomeric species is also featured in the sedimentation velocity profiles of *Bacillus subtilis* Lon^25^.

Our structural and mutational analyses also suggest that the assembly of the pentameric oligomer and the allosteric structural change induced by the binding of the sixth protomer are mediated by the THT, which non-covalently bridges together the substrate-binding NGDs domains and the AAA+ domains of the three Ob-protomers Ob1, Ob3, and Ob5. Interaction of substrate protein with three inner NDG domains likely triggers the structural changes of the three tethered Ob-protomers, allowing the incorporation of the sixth protomer Ac6* to form the well-known close-ring structure with the right-handed staircase arrangement of the substrate-pore-loop contacts (Fig. 6).

To corroborate the proposed activation model, we have constructed the monomeric mutants, E613K and L708R, of MtaLon and the equivalent E614K and L709R of EcoLon, which lack the N-terminal NGD and LH regions. Our results show the binding of truncated monomeric mutants to the pentameric form (Fig. 4c, d). Also, we show that full-length Lon incubated with a monomeric mutant assembles into not only the expected *5*+*1* heterocomplex but also the spiral pentamers and spiral hexamers (Supplementary Fig. 13). Despite the presence of the native spiral hexamers, monomeric mutants are able to completely inhibit substrate degradation by wild-type Lon, in a concentration-dependent manner (Fig. 4e). These results suggest that the spiral pentameric form, rather than the spiral hexameric form, is a key mediator for the substrate-dependent activation of Lon. The pentamer-mediated activation model provides the missing mechanistic link between the two previously known conformational states of AAA+ proteins: namely, the substrate-free left-handed hexameric spiral and the substrate-bound close-ring hexamer with a right-handed spiral arrangement. Coincidentally, one of the monomeric mutations we designed for this study is identical to the naturally occurring *CapR9* mutation of EcoLon found in the early 1980s, which displayed a genetically unusual phenotypic dominance^26^. The CapR9 protein was found to be proteolytically inactive, prone to disassembly, and yet was able to inhibit wild-type EcoLon activity^27,28^. These earlier findings lend further support to the proposed activation model.

In conclusion, these findings point to a unique paradigm for the activation mechanism of a AAA+ protease, which may be induced by a substrate-controlled assembly process involving specifically a pentameric assembly. Our proposed model highlights how an intracellular proteolytic machine, which is potentially destructive if uncontrolled, is tightly governed by the presence of a substrate. Finally, this work also showcases a strategy for inhibiting a specific AAA+ protein within cells, thereby impeding the formation of persister cells of *E. coli*. These results may have the potential to open up an avenue for targeted inhibition of specific AAA+ proteins in the cell for therapeutic applications.

## Methods

### Cloning and mutagenesis

The full-length *Meiothermus taiwanensis* LonA (MtaLon) cloned into the bacterial expression vector pET21a(+) with a C-terminal 6 × His tag, as well as single mutants Y224S, S678A, M217A, and Y397A, have been described previously^19^. The N-terminal domain (residues 1–241) removed MtaLon (ΔN) cloned into pET28a(+) with a tobacco etch virus (TEV) cleavage site has been described previously^29^; single mutants of ΔN-E613K and ΔN-L708R were performed by site-directed mutagenesis. *Escherichia coli* Lon (EcoLon) without the N-terminal domain (residues 1–246)(EcoLon-ΔN) was cloned into the bacterial expression vector pBAD18 using Gibson Assembly® (NEB), and single mutants of ΔN-E614K and ΔN-L709R were performed by site-directed mutagenesis. Sequences of the primers are listed in the Source Data file. All constructs in this study were verified by the DNA Sequencing Core Facility of Academia Sinica.

### Protein expression and purification

Plasmid-transformed BL21(DE3) cells were plated onto agar plates containing LB/carbenicillin (CBC, 50 μg/mL), and incubated overnight at 37 °C. A single colony was added into 1 liter LB medium containing antibiotics, and incubated at 37 °C and 250 rpm until the OD_600_ reached 0.6 to 0.8. Isopropyl β-d-thiogalactopyranoside (IPTG) was added to the cell culture at 1 mM working concentration, and the cell culture was incubated for another 4 h at 25 °C and 150 rpm^29,30^. Cells were harvested by centrifugation at 6000 rpm (JLA-8.1000 rotor, Beckman) for 20 min at 4 °C and cell pellets were suspended in the lysis buffer (50 mM Tris-HCl pH 8.0 and 500 mM NaCl). The cell lysate was first ruptured by a French press (Avestin) at 15,000–20,000 psi and centrifuged at 40,000 rpm (Type 45 Ti rotor, Beckman) for 1 h at 4 °C. The supernatant was incubated with Ni-nitrilotriacetic acid (Ni-NTA) resins (Qiagen) at 4 °C for 2 h. The resins were further washed with an imidazole gradient (0–40 mM) and eluted with 250 mM imidazole. The eluted proteins were concentrated and subjected to size-exclusion chromatography (SEC) on a Superose 6 Increase (GE Healthcare) column pre-equilibrated with 20 mM Tris-HCl (pH 8.0), 150 mM NaCl, 10 mM MgCl_2_, and 1 mM DTT. Overexpression of EcoLon-ΔN-E614K and ΔN-L709R in MG1655 cells, induced by 0.5% L-arabinose at 20 °C for 16 h, was confirmed by whole-cell Western blot analysis using a 6x-His tag monoclonal antibody at a 1:10,000 dilution (*clone* HIS.H8, Abcam ab18184), and further evaluated by Ni-NTA chromatography with production yields of 19.2 and 21.1 mg/L of culture, respectively (Supplementary Fig. 14). The anti-*E. coli* RNA Polymerase β (rpoB) antibody (clone 8RB13, Biolegend 663903, dilution 1:10,000) was used for an internal loading control.

### Fluorescence polarization (FP)

MtaLon samples were serially diluted (from 5 µM to 0.01 µM) and mixed with 1.25 µM FITC-casein in the presence of 1 mM ADP and incubated at 4 °C for 3 h. Detection of fluorescence polarization values (mP) was performed on a multifunctional microplate reader (Infinite M1000 pro, Tecan) in triplicates; the wavelengths 470 nm (excitation) and 520 nm (emission) were used for measurement. The FP values (mP) were calculated according to the following equation:

$${FP}=1000\times \frac{{I}^{{Par}}-G\times {I}^{{Per}}}{{I}^{{Par}}+G\times {I}^{{Per}}}$$

Where *I*^*Par*^ is the parallel emission intensity, *I*^*Per*^ is the perpendicular emission intensity, and *G* is grating factor. The value of the G-factor was set to 0.98 after calculation by Infinite M1000 pro. The FP values of all samples are shown as ΔFP, from which the control value was subtracted, and plotted against the protein concentrations. All ΔFP values are presented as mean and SD (*n* = 3).

### Cryo-EM grid preparation and data acquisition

All of the purified MtaLon proteins were diluted to a final concentration of 1 μM (calculated as hexamer). Three microliters of the sample were applied onto glow-discharged 200-mesh R1.2/1.3 Quantifoil copper grids. The grids were blotted for 2.5 to 4 s and rapidly cryocooled in liquid ethane using a Vitrobot Mark IV (Thermo Fisher Scientific) at 4 °C and 100% humidity. For the specimens of MtaLon:ADP, MtaLon-Y224S:ATPγS, and S678A:casein:ADP, casein and/or nucleotides (ADP or ATPγS) were added in a final concentration of 10 μM (casein) and/or 5 mM (nucleotides), and incubated overnight prior to cryo-grid preparation. For MtaLon-Y397A/S678A:casein:ATPγS and MtaLon-M217A:casein:ADP, casein and nucleotides were added in a final concentration of 10 μM (casein) and 5 mM (nucleotides), and incubated for 3 h prior to cryo-grid preparation. For the *5*+*1* heteromeric complex, 10 μM ΔN-E613K and 5 mM ADP were incubated with 1 μM MtaLon-Y224S at 4 °C for 1 h, and the MtaLon-Y224S:ΔN-E613K:ADP mixture was purified on a Superose 6 Increase (GE Healthcare) column prior to cryo-grid preparation. The details for data collection are listed in Supplementary Tables 1–7.

### Cryo-EM data processing

For the MtaLon-Apo and MtaLon:ADP data, micrographs were first imported into Relion^31^ for image processing, followed by motion-correction performed using MotionCor2^32^, and determining the contrast transfer function (CTF) using CTFFIND4^33^. The micrographs with “rlnMotionEarly <10” and “rlnCtfMaxResolution <5” were selected using the “subset selection” option in Relion. All particles were autopicked using the NeuralNet option (threshold 1 = 0; threshold 2 = −5) in EMAN2^34^. For the data of MtaLon-Y224S:ATPγS and S678A:casein:ADP, micrographs were imported into cryoSPARC (Version: 3.2) and processed using patch motion correction and patch CTF estimation. The output F-crop factor was set to ½ to crop the pixel size to 1.061 Å. For the data sets of Y397A/S678A:casein:ATPγS, MtaLon-M217A:casein:ADP, and MtaLon-Y224S:ΔN-E613K:ADP, on-the-fly motion correction with dose-weighting was implemented during the image acquisition by MotionCor2^32^, with a 7 × 5 patch and a two-fold binning, and the CTF was determined by patch CTF estimation in cryoSPARC. All particles were autopicked using the template picker in cryoSPARC (Version: 3.2). After 2D classification, particles in good 2D classes were used for ab initio map generation, followed by 3D heterogeneous refinement without imposing symmetry (C1). The particles belonging to the good 3D classes were then refined into higher resolutions by homogeneous, non-uniform, and local refinements. Resolutions for the final maps were estimated with the 0.143 criterion of the Fourier shell correlation curve. Resolution maps were calculated in cryoSPARC using the “Local Resolution Estimation” option. The processing details are shown in Supplementary Figs. 2–4, 6, 9–10 and 13, and listed in Supplementary Tables 1–7.

### Model building and refinement

Model building was first performed on wild-type MtaLon incubated with ADP. The full-length sequence of MtaLon was imported into the SWISS-MODEL server^35^ for initial model generation based on the coordinates of PDB code 6U5Z^24^, resulting in a spirally arranged hexamer containing the AAA+ and protease domains (residues 243–774).

For the 5.8-Å trimer or 3.8-Å tetramer maps without the N-terminal domain, the corresponding number of protomers from the above initial model were rigidly fitted into the cryo-EM map, and then molecular dynamics flexible fitting (MDFF)^36^ was used to flexibly fit the atomic model into the map. The resultant model (residues 243–774) was optimized by Coot^37^ and refined using phenix.real_space_refine^38^.

For the 3.6-Å pentamer or 3.8-Å hexamer maps containing the N-terminal domain, the core region (residues 243–774) was modeled as described for building the trimer and tetramer structures; the N-terminal domain (residues 1–237) (PDB code 7FD4) from our previous study was rigidly fitted into the cryo-EM map, followed by MDFF; the core region and the N-terminal domain were combined, and the linker (residues 238–242) between the N-terminal domain and the AAA+ domain was manually built using Coot to get the full-length model. Then phenix.real_space_refine was applied for model optimization.

The modeling procedures for the apo and mutant MtaLon in the spiral oligomeric forms were carried out using the coordinates of the corresponding oligomeric structures of MtaLon:ADP as the initial models, and the final models were refined using phenix.real_space_refine, and by manual rebuilding using Coot. To build the substrate-bound close-ring hexameric structure, the coordinate of PDB code 7FID was used as the starting model. The types of the bound nucleotides were determined by LigandFit in Phenix, with an overall correlation coefficient of the ligand to the map over 0.7.

To construct the “*5*+*1*” heterohexameric complex, the spiral hexamer of MtaLon:ADP was utilized as the initial model to build the core region (residues 243–774); the N-terminal domain (residues 1–237) was rigidly fitted into the cryo-EM map by manual adjustments using ChimeraX. The combination of core region and the N-terminal domain was manually built using Coot as previously described. Then the model was optimized by Coot and phenix.real_space_refine. The final models were evaluated by MolProbity^39^. The map-model correlation coefficients, CC_box_, were calculated by phenix.map_model_cc^40^. The statistics of the map reconstruction and model building are summarized in Supplementary Tables 1–7. All figures were prepared using ChimeraX^41^ and PyMol^42^.

### Protein labeling

MtaLon-ΔN-E613K and ΔN-L708R proteins were exchanged to a buffer solution of 20 mM HEPES, pH 7.5, 200 mM NaCl, 10 mM MgCl_2_ buffer, and diluted to 20 μM for labeling. Then, 100 μL diluted protein was mixed with 1 μL, 10 mM Alexa Fluor 488 NHS Ester (Thermo Fisher Scientific), and incubated at room temperature for 1 h. Desalting was carried out to remove excess free dye by using a PD MiniTrap G-25 column (Cytiva). The label efficiency of ΔN-E613K and ΔN-L708R is 80% determined through UV-Vis spectroscopy (A280 and A496 measurements) on a DU730 UV-Vis spectrometer (Beckman Coulter). Alexa Fluor 488-labeled ΔN-E613K and ΔN-L708R alone (3.3 μM) or each incubated with MtaLon_6_ in a 1:2 molar ratio (3.3 μM and 6.6 μM) were studied by SV-AUC and SEC-MALS (see the next two sections).

### Sedimentation velocity analytical ultracentrifugation (SV-AUC)

AUC experiments were performed using an XL-A analytical ultracentrifuge equipped with an An-60 Ti rotor (Beckman Coulter). Then, 400 μL samples (see above) and 406 μL buffer (20 mM HEPES, pH 7.5, 200 mM NaCl, 10 mM MgCl_2_) were loaded into a 12 mm double sector charcoal-filled epon centerpiece (Beckman Coulter) and centrifuged at 20 °C at a speed of 40,000 rpm for 16 h. The absorbance of the samples at 490 nm was monitored in a continuous mode at time intervals of 5 min. The partial specific volume, buffer density, and viscosity were calculated by SEDNTERP. Data were analyzed with the standard c(s) method in Sedfit 16.1c; the diffusion deconvoluted sedimentation coefficient distributions were normalized by total integrated area and derived from data acquired at 490 nm.

### Size-exclusion chromatography coupled to multi-angle light scattering (SEC-MALS)

SEC–MALS measurements were carried out with a miniDAWN TREOS detector (Wyatt Technology Corporation) coupled to an Agilent 1260 Infinity HPLC. Then, 100 μL samples (see above) were injected into a size-exclusion chromatography column (ENrich SEC 650, Bio-Rad) which was pre-equilibrated with the running buffer (20 mM HEPES, pH 7.5, 200 mM NaCl, 10 mM MgCl_2_ and 0.02% NaN_3_). A constant flow rate of 0.5 mL/min was used, and UV absorbance was monitored simultaneously at 280 nm and 488 nm. Molecular weights were determined by multi-angle laser light scattering using an in-line miniDAWN TREOS detector and an Optilab T-rEX differential refractive index detector (Wyatt Technology Corporation). Bovine serum albumin (Sigma, A1900) was used for system calibration, and the data were analyzed using ASTRA 6 software (Wyatt Technology Corporation) with the dn/dc value set to 0.185 mL/g, and plotted by GraphPad Prism 9.1.2 (GraphPad Software, LLC).

### Western blot analysis of size-exclusion chromatography

MtaLon-WT (20 μM, hexamer concentration) was incubated with 15 μM of either MtaLon-ΔN-E613K or -L708R in a buffer containing 20 mM Tris-HCl (pH 8.0), 200 mM NaCl, and 10 mM MgCl_2_ at room temperature overnight. Subsequently, 50 μL of the reaction mixtures were applied to a Superdex 200 Increase column (Cytiva), and eluted fractions were collected for Western blot analysis. SDS-PAGE was performed at 120 V for 90 min, and proteins were transferred to a nitrocellulose membrane at 90 V for 1 h at 4 °C. The membrane was blocked with 5% non-fat milk for 1 h at room temperature and then incubated overnight at 4 °C with a 6x-His tag monoclonal antibody (clone HIS.H8, Abcam ab18184) at a 1:10,000 dilution for the detection of EcoLon-WT, ΔN-E614K, and ΔN-L709R. Afterward, the membrane was washed four times for 5 min each with PBS containing 0.1% Tween 20 (PBST) and incubated with the horseradish peroxidase (HRP)-conjugated anti-mouse IgG antibody (Abcam ab6789) at a 1:20,000 dilution for 1 h at room temperature. Excess secondary antibody was removed by washing with PBST. Proteins were detected using the Immobilon Western Chemiluminescent HRP substrate (Merck Millipore), and images were acquired using a LAS-4000 mini imaging system (Fujifilm).

### Substrate degradation assay

MtaLon-WT (0.5 μM, calculated as hexamer) was incubated with MtaLon-ΔN-E613K or ΔN-L708R  (0–20 μM) in the reaction buffer containing 20 mM Tris-HCl (pH 8.0), 200 mM NaCl, and 10 mM MgCl_2_ at room temperature for 1 hour, then α-casein (10 μM) and ATP (5 mM) were added to the reaction mixture (total 20 μL) and incubated at 55°C for 15 minutes. Reactions were stopped by adding 5 μL 5× gel-loading dye in a 20 μL reaction mixture and heating at 95°C for 5 minutes. Substrate degradation was assessed by SDS-PAGE and Coomassie Blue staining.

### Colony morphology

*E. coli* MG1655 cells transformed with pBAD18-EcoLon-ΔN-E614K or -ΔN-L709R were incubated on LB agar plates with 50 μg/mL CBC at 37 °C overnight. A single colony was selected and suspended in 50 μL of LB broth, followed by streaking onto an LB agar plate containing CBC and 0.5% L-arabinose. The plate was incubated at room temperature for 36 h.

### Cell imaging

*E. coli* MG1655 cells transformed with EcoLon-ΔN-E614K and -ΔN-L709R were induced with 0.5% L-arabinose at 20 °C and 150 rpm overnight. The OD_600_ values of overnight culture were adjusted to 0.2 with LB containing antibiotic and 0.5% L-arabinose, and irradiated with 10 mJ/cm^2^ at 254 nm. The irradiated cells were subsequently incubated at 37 °C and 250 rpm for 3 h. After incubation, 1 μL of each culture was spotted on agarose pads (2% w/v in PBS, 1 mm thick). Phase contrast microscopy was performed on the Olympus scanR high-content screen station with an Olympus UPLXAPO60XOPH 60x (1.42 NA) oil-immersion objective. Cell area was analyzed by ImageJ with the plug-in MicrobeJ^43^.

### UV sensitivity and persister formation assays

*E. coli* MG1655 cells were transformed with pBAD18-EcoLon-ΔN-E614K or -ΔN-L709R, and incubated on LB agar plates with 50 μg/mL CBC at 37 °C overnight. To prepare preculture, the colony was inoculated into 1 mL LB with CBC and incubated at 37 °C and 250 rpm overnight. To induce protein expression, preculture was added into 20 mL LB with CBC and expanded to mid-exponential phase (OD_600_ ≅ 0.4) at 37 °C and 250 rpm, then induced with 0.5% L-arabinose at 20 °C and 150 rpm overnight. The overnight culture was diluted with phosphate-buffered saline (PBS) to an OD_600_ of 0.2 and irradiated by a Stratalinker UV Crosslinker with 0.5, 1, 2, 5, and 10 mJ/cm^2^ at 254 nm. To investigate persister cell formation, the overnight culture was adjusted to an OD_600_ of 0.2 with LB containing CBC and 0.5% L-arabinose and treated with 5 μg/mL ofloxacin (OFX) for 1, 2, 4, 6, and 24 h. 1 mL of OFX-treated cells was collected at the indicated time and centrifuged at 13,000 rpm for 3 min. After removal of the supernatant, the cell pellet was washed twice with 1 mL PBS and resuspended in 100 μL PBS. Colony-forming unit (CFU) enumeration was performed to evaluate the viability of the irradiated and OFX-treated cells; the treated cells were subjected to serial dilution (up to 10^−6^), and 10 μL of each dilution was spotted onto LB agar plates containing CBC and 0.5% L-arabinose. The plates were then incubated at 37 °C for 16 h.

### Statistical analysis

In vivo experiments were performed with a minimum of three independent biological replicates. Data analysis was conducted using GraphPad Prism 9.5.1, and statistical significance was assessed using one-way analysis of variance (ANOVA) with Tukey’s multiple comparisons test. *P*-values were calculated to evaluate the statistical significance of the estimated differences.

### Reporting summary

Further information on research design is available in the Nature Portfolio Reporting Summary linked to this article.

### Supplementary information

Supplementary Information

Peer Review File

Reporting Summary

### Source data

Source Data

## Data Availability

The accession numbers of the cryo-EM maps of MtaLon-Y224S:ATPγS, deposited in the Electron Microscopy Data Bank (EMDB), for the spiral pentamer and spiral hexamer are EMD-3 4000 and EMD- 34001, respectively; the coordinates of the atomic models for the spiral pentamer and spiral hexamer are deposited in the Protein Data Bank (PDB) with the PDB codes 7YPH [10.2210/pdb7YPH/pdb] and 7YPI [10.2210/pdb7YPI/pdb], respectively. The accession numbers of the cryo-EM maps of MtaLon-Apo for the spiral oligomers of trimer, tetramer, pentamer, and hexamer are EMD- 34107, EMD- 34108, EMD- 34109, and EMD- 34110, respectively; the PDB codes for the spiral oligomers of trimer, tetramer, pentamer, and hexamer are 7YUH [10.2210/pdb7YUH/pdb], 7YUM [10.2210/pdb7YUM/pdb], 7YUP [10.2210/pdb7YUP/pdb], and 7YUT [10.2210/pdb7YUT/pdb], respectively. The accession numbers of the cryo-EM maps of MtaLon:ADP for the spiral oligomers of trimer, tetramer, pentamer, and hexamer are EMD- 34111, EMD- 34112, EMD- 34113, and EMD- 34114, respectively; the PDB codes for the spiral oligomers of trimer, tetramer, pentamer, and hexamer are 7YUU [10.2210/pdb7YUU/pdb], 7YUV [10.2210/pdb7YUV/pdb], 7YUW [10.2210/pdb7YUW/pdb], and 7YUX [10.2210/pdb7YUX/pdb], respectively. The accession numbers of the cryo-EM maps of MtaLon-S678A:casein:ADP for the spiral pentamer, spiral hexamer, and close-ring hexamer are EMD- 34002, EMD- 34004, and EMD- 34003, respectively; the PDB codes for the spiral pentamer and close-ring hexamer are 7YPJ [10.2210/pdb7YPJ/pdb] and 7YPK [10.2210/pdb7YPK/pdb], respectively. The accession numbers of the cryo-EM maps of MtaLon-Y397A/S678A:casein:ATPγS for the spiral pentamer and spiral hexamer are EMD- 34005 and EMD- 34006, respectively. The accession numbers of the cryo-EM maps of MtaLon-M217A:casein:ADP for the spiral pentamer and spiral hexamer are EMD- 34116 and EMD- 34117, respectively. The accession numbers of the cryo-EM maps of MtaLon-Y224S:ΔN-E613K:ADP for the spiral pentamer, spiral hexamer, and the *5*+*1* heterocomplex are EMD- 36865, EMD- 36866, and EMD- 36867, respectively; the PDB code for the *5*+*1* heterocomplex is 8K3Y [10.2210/pdb8K3Y/pdb]. Source data are provided with this paper.
