# Supplementary material for: A 5+1 assemble-to-activate mechanism of the Lon proteolytic machine
Source: Nat Commun. 2023 Nov 13;14:7340. doi: 10.1038/s41467-023-43035-2 (PMC10643698; doi:10.1038/s41467-023-43035-2)
Supplement: Supplementary file 1 — Supplementary Information [file 41467_2023_43035_MOESM1_ESM.pdf]

Supplementary Information

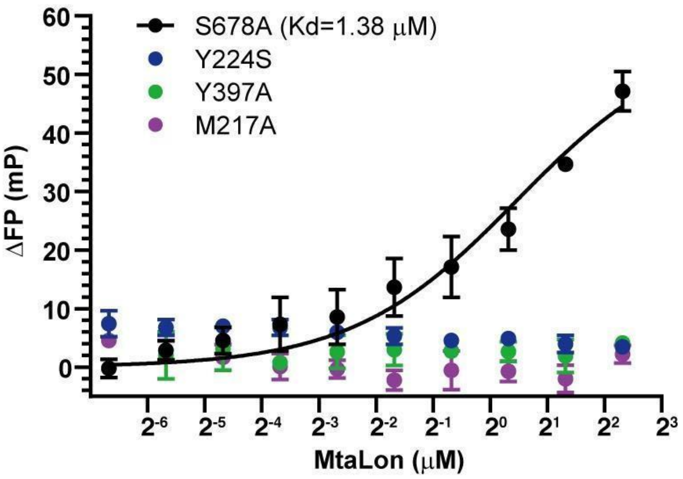

Supplementary Fig. 1: Identification of MtaLon mutants with no substrate-binding activity. Fluorescence polarization (FP) of the interaction between FITC-casein and MtaLon proteins. Changes of FP of FITC-casein upon addition of various MtaLon mutants were plotted in different concentrations. mP, unit for FP. Data are presented as mean with SD (as shown by error bars) of three independent experiments (n = 3). Source data are provided as a Source Data file.

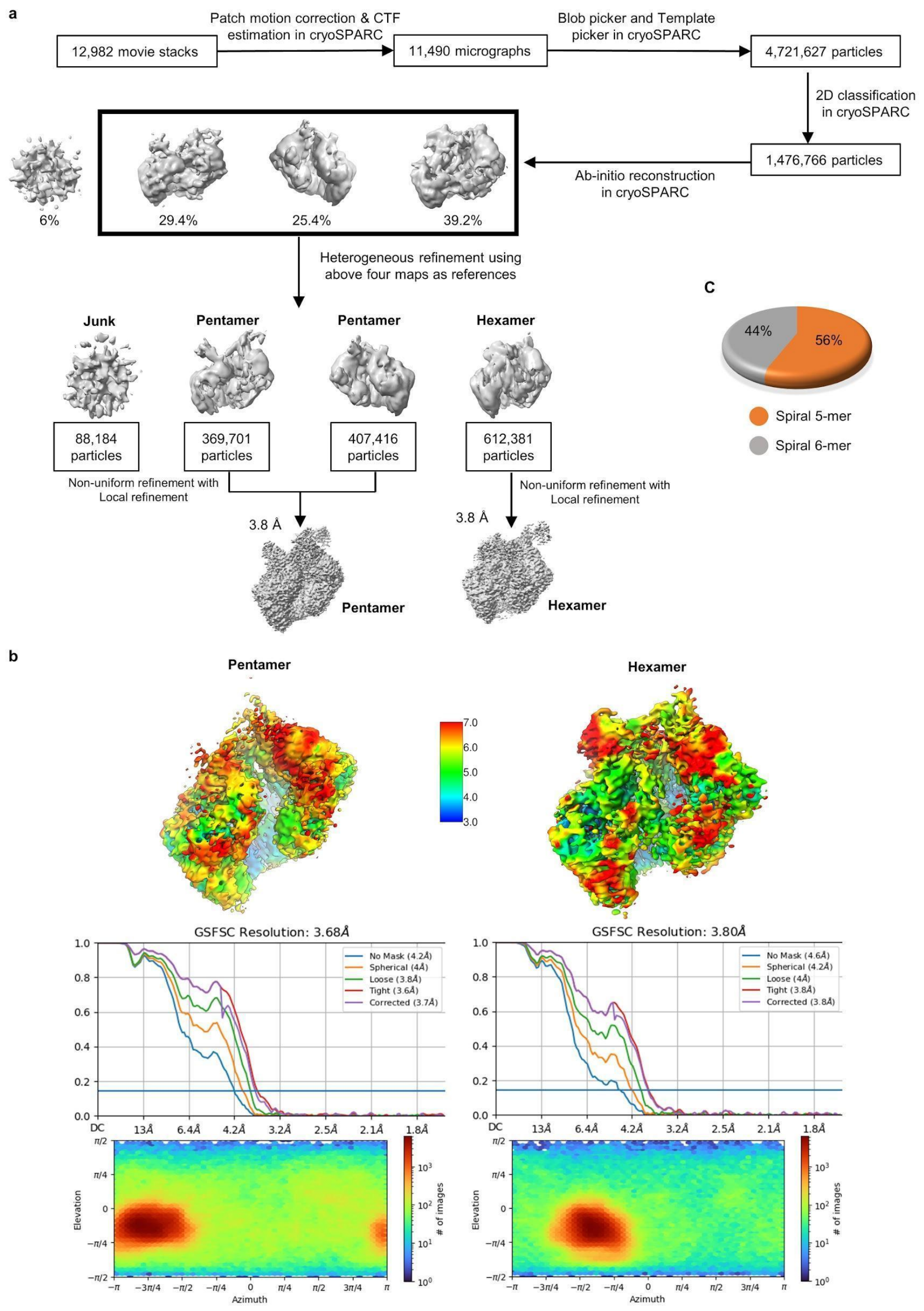

Supplementary Fig. 2: Single-particle cryo-EM analysis of MtaLon-Y224S:ATPγS. a, Workflow of the data processing. **b,** Evaluation of the final 3D reconstructions. Top: Resolution maps for the final 3D reconstructions; middle: Gold standard FSC plots for the final 3D reconstructions, calculated in cryoSPARC; bottom: Euler angle distribution of the particle images. **c,** Pie chart showing the ratio of spiral penamers and spiral hexamers.

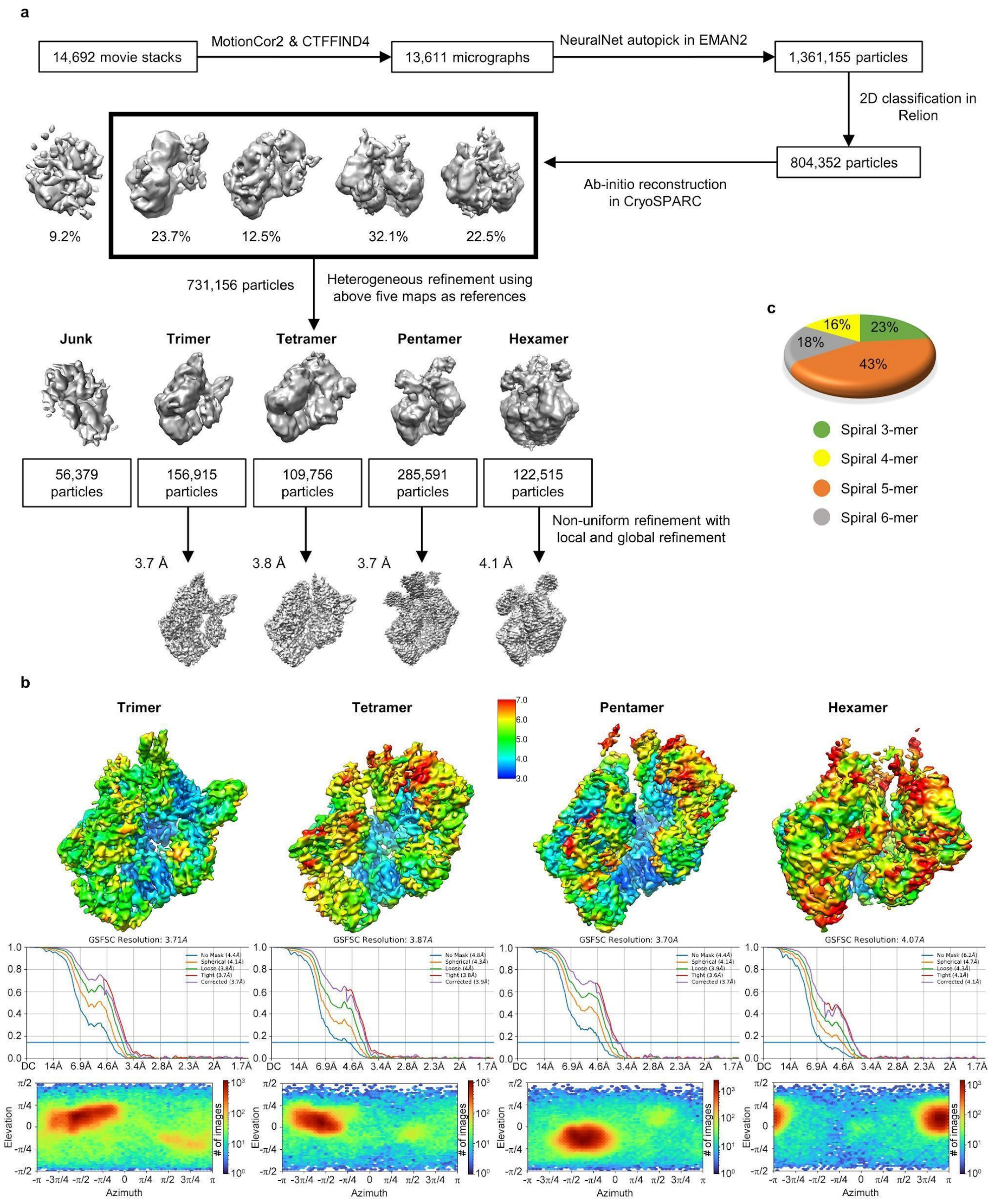

Supplementary Fig. 3: Single-particle cryo-EM analysis of MtaLon-Apo. a, Workflow of the data processing. **b,** Evaluation of the final 3D reconstructions. Top: Resolution maps for the final 3D reconstructions; middle: Gold standard FSC plots for the final 3D reconstructions, calculated in cryoSPARC; bottom: Euler angle distribution of the particle images. **c,** Pie chart showing the ratio of different spiral oligomers.

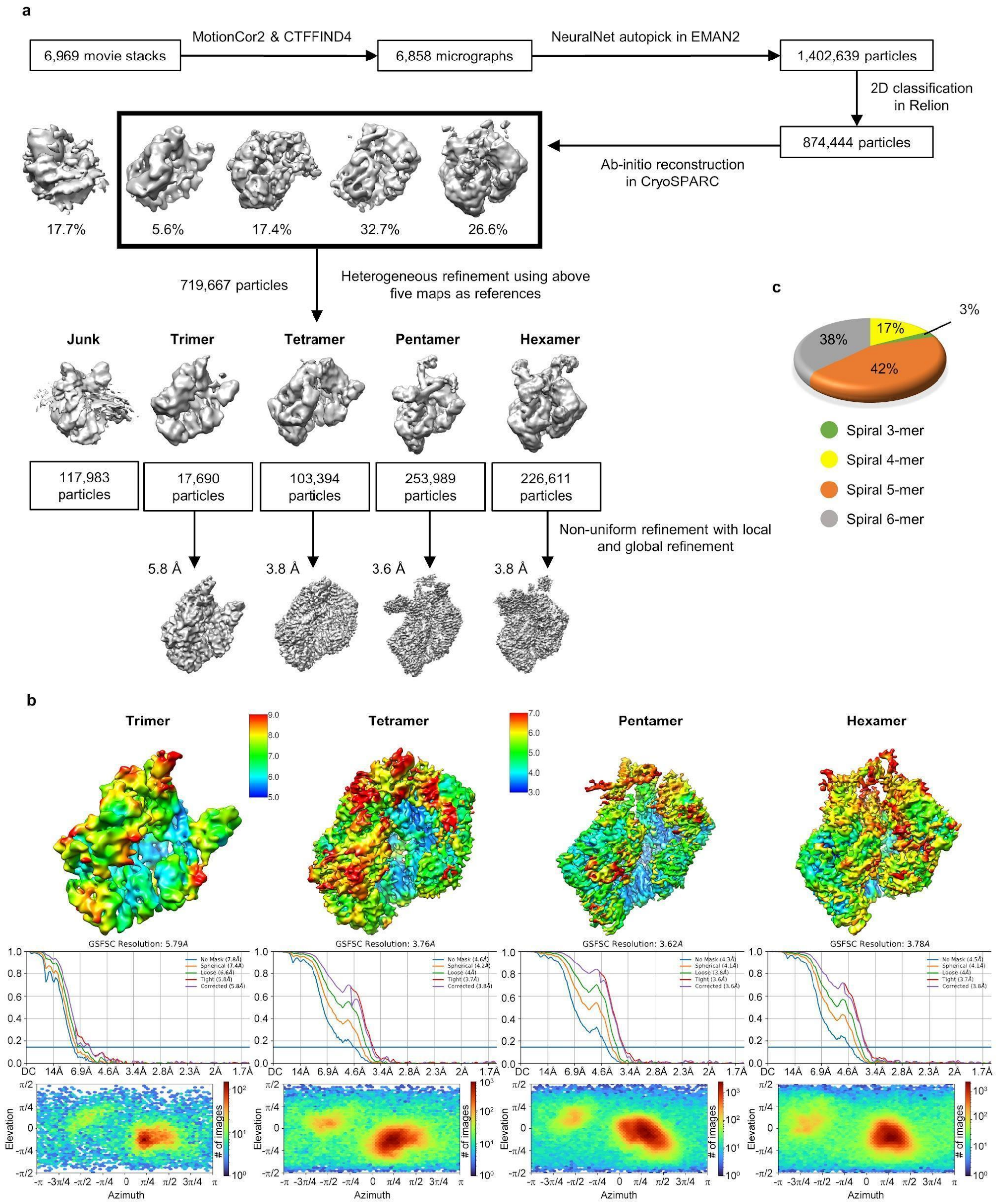

Supplementary Fig. 4: Single-particle cryo-EM analysis of MtaLon:ADP. a, Workflow of the data processing. **b,** Evaluation of the final 3D reconstructions. Top: Resolution maps for the final 3D reconstructions, with the color bar shared by all maps except the trimer; middle: Gold standard FSC plots for the final 3D reconstructions, calculated in cryoSPARC; bottom: Euler angle distribution of the particle images. **c,** Pie chart showing the ratio of different spiral oligomers.

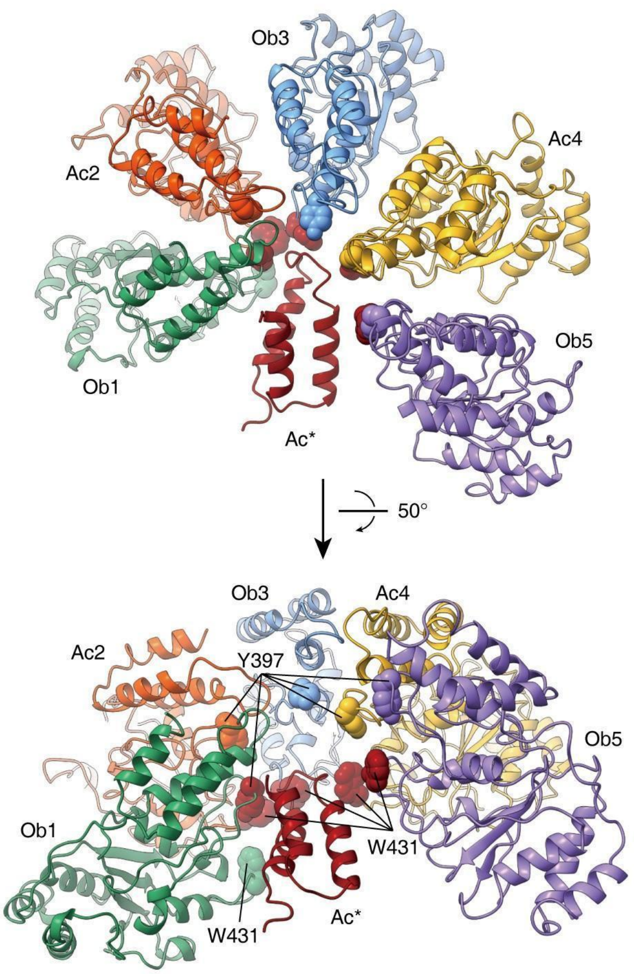

Supplementary Fig. 5: The substrate-free spiral hexamer forms an autoinhibited conformation. Two views of the spiral hexamer are shown in ribbon models. For clarity, the NGDs, LHs, and protease domains were omitted. The 3H subdomain of Ac6* (ribbon in dark red) is shown to occupy the axial position of the spiral organization of five AAA+ domains from protomers 1~5. The pore-loop-I residue Y397 and the pore-loop-II residue W431 are shown in spheres; those whose solvent accessibility is blocked by the 3H subdomain of Ac6* are colored in dark red.

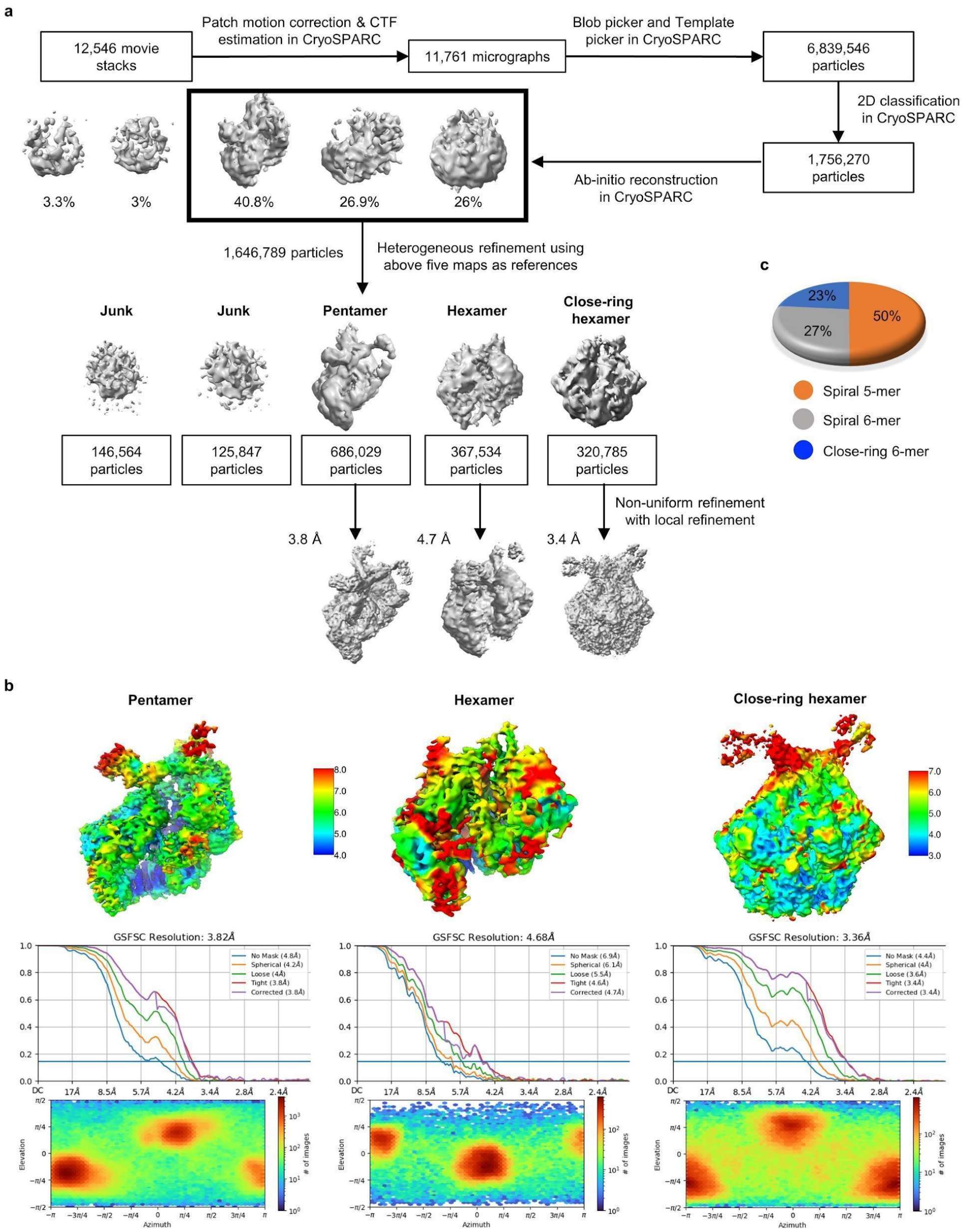

Supplementary Fig. 6: Single-particle cryo-EM analysis of MtaLon-S678A:casein:ADP. a, Workflow of the data processing. **b,** Evaluation of the final 3D reconstructions. Top: Resolution maps for the final 3D reconstructions, with the color bar shared by all maps except the close-ring hexamer; middle: Gold standard FSC plots for the final 3D reconstructions, calculated in cryoSPARC; bottom: Euler angle distribution of the particle images. **c,** Pie chart showing the ratio of spiral pentamers, spiral hexamers, and close-ring hexamers.

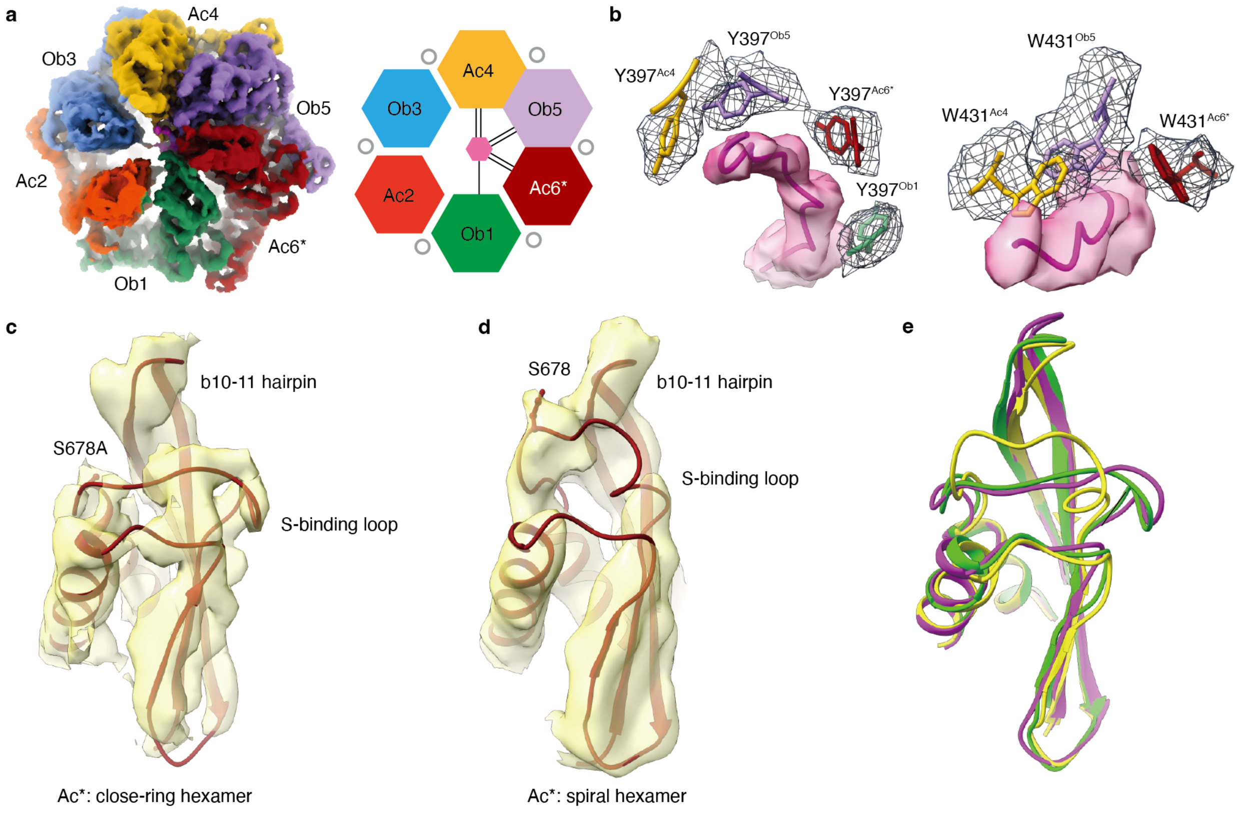

Supplementary Fig. 7: Structure of the close-ring hexamer of MtaLon-S678A bound to substrate and ADP. a, Top view of the map (left) and cartoon diagram (right) of the close-ring hexamer. The N-terminal regions were omitted for clarity. Gray open circles represent ADP. **b,** Close-up top view of the substrate and the contacting pore-loop residues fit to the map, shown in the same color scheme as (**a**). **c,d,** Coil models fit to the maps of the proteolytic active sites of the protomers Ac6* in the substrate-bound close-ring hexamer of MtaLon-S678A:casein-ADP (**c**) and in the substrate-free spiral hexamer of MtaLon-ADP (**d**). **e,** Superimposition of the proteolytic sites of the protomers Ac6*, shown in (**c**) and (**d**) (colored in green and yellow, respectively), with the bortezomib-bound proteolytic site of Ac6* in the close-ring structure of MtaLon:casein-ATPγS (colored in purple; PDB code 7FD4, chain C; bortezomib omitted for clarity).

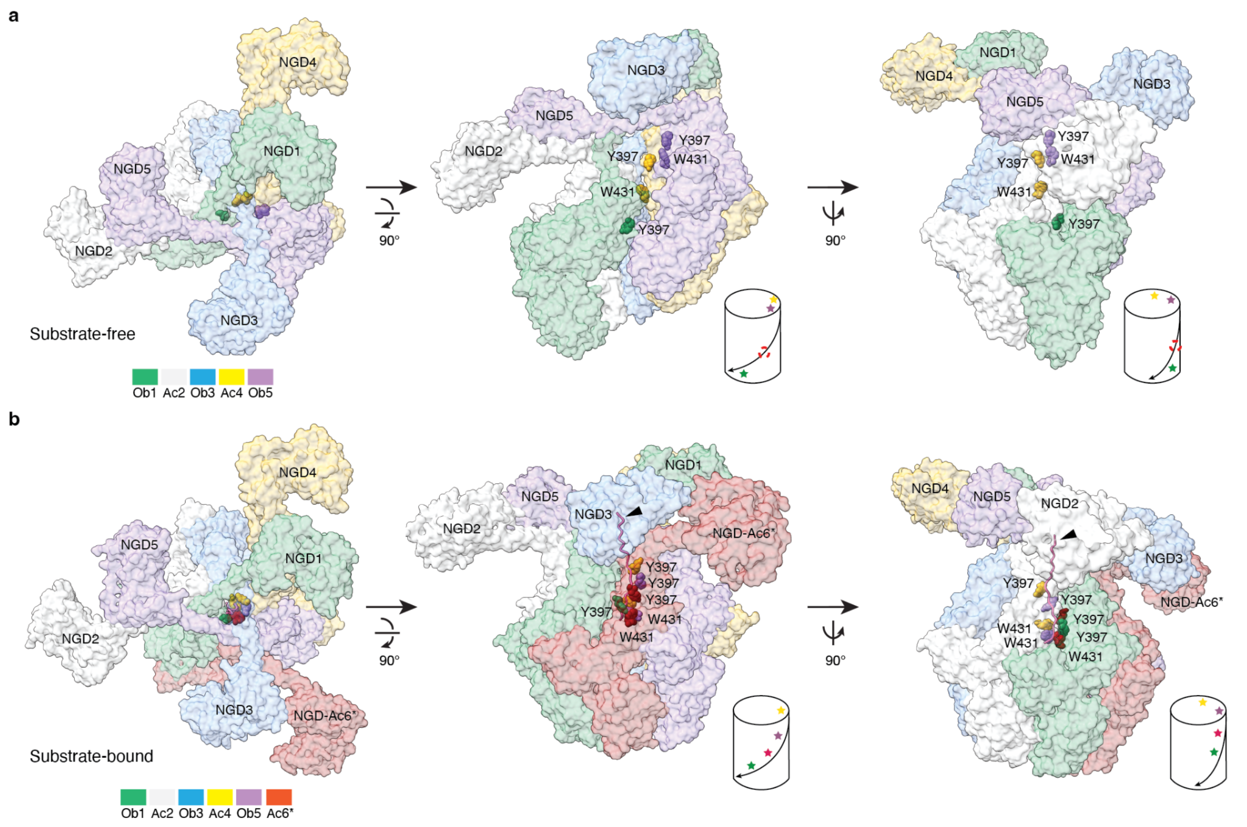

Supplementary Fig. 8: Distribution of the pore-loop residues in the substrate-free and substrate-bound states of MtaLon. a, The substrate-free pentameric structure is shown in surface representation with 80% transparency. The pore-loop I and II residues (Y397 and W431, respectively) are shown in spheres and colored using the indicated coloring scheme. The cylindrical cartoon illustrates a spiral right-handed trajectory; the star symbols denote the pore-loop-I residues. The dotted circle indicates where Y397 of the sixth protomer Ac6* may line up on the trajectory upon binding. **b**, The substrate-bound hexameric form of MtaLon-S678A incubated with ADP and the substrate α-casein is shown in surface representation with 80% transparency. The substrate polypeptide chain is marked by the arrowhead.

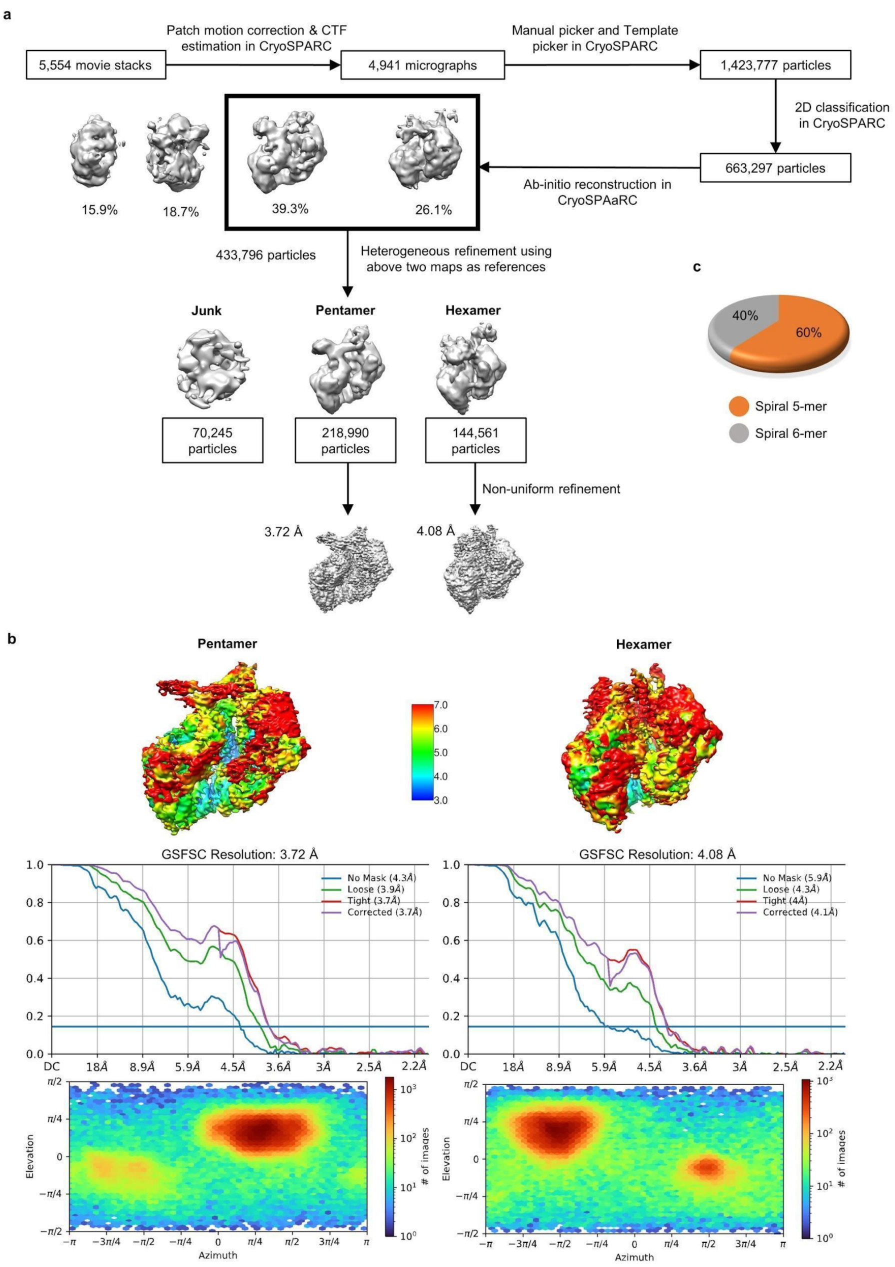

Supplementary Fig. 9: Single-particle cryo-EM analysis of MtaLon-Y397A/S678A: casein:ATPγS. a, Workflow of the data processing. **b,** Evaluation of the final 3D reconstructions. Top: Resolution maps for the final 3D reconstructions; middle: Gold standard FSC plots for the final 3D reconstructions, calculated in cryoSPARC; bottom: Euler angle distribution of the particle images. **c,** Pie chart showing the ratio of spiral penamers and spiral hexamers.

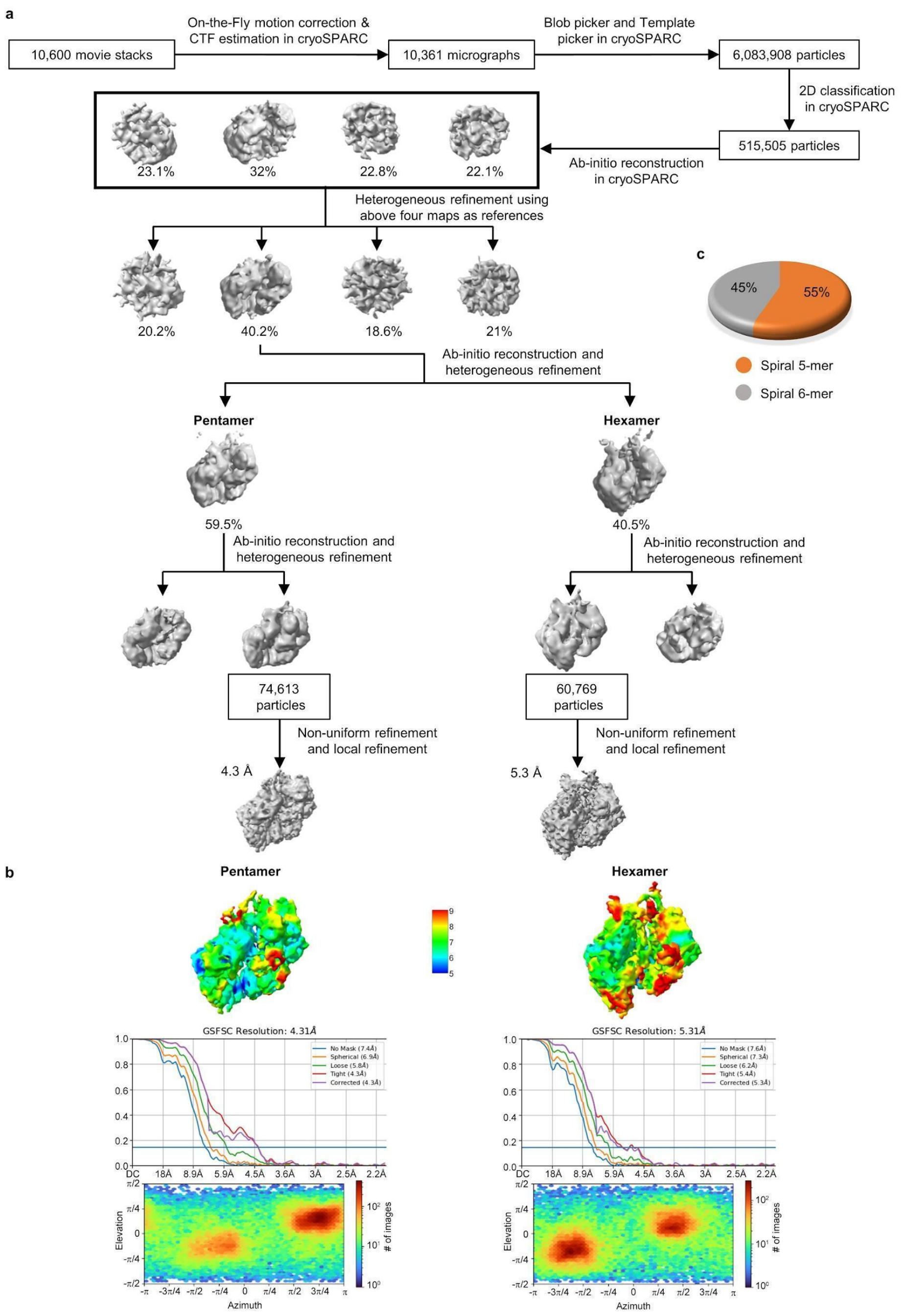

Supplementary Fig. 10: Single-particle cryo-EM analysis of MtaLon-M217A:casein:ADP. **a,** Workflow of the data processing. **b,** Evaluation of the final 3D reconstructions. Top: Resolution maps for the final 3D reconstructions; middle: Gold standard FSC plots for the final 3D reconstructions, calculated in cryoSPARC; bottom: Euler angle distribution of the particle images. **c,** Pie chart showing the ratio of spiral penamers and spiral hexamers.

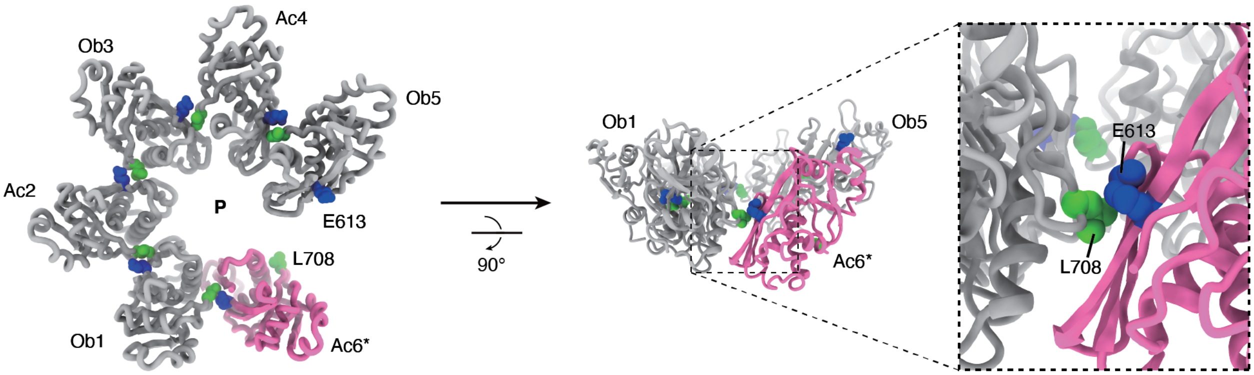

Supplementary Fig. 11: Interface of the protease domains of MtaLon. Two views showing the protease domains (P) in the spiral hexamer in ribbons. The interacting residues Glu613 (blue) and Leu708 (green) are highlighted in spheres.

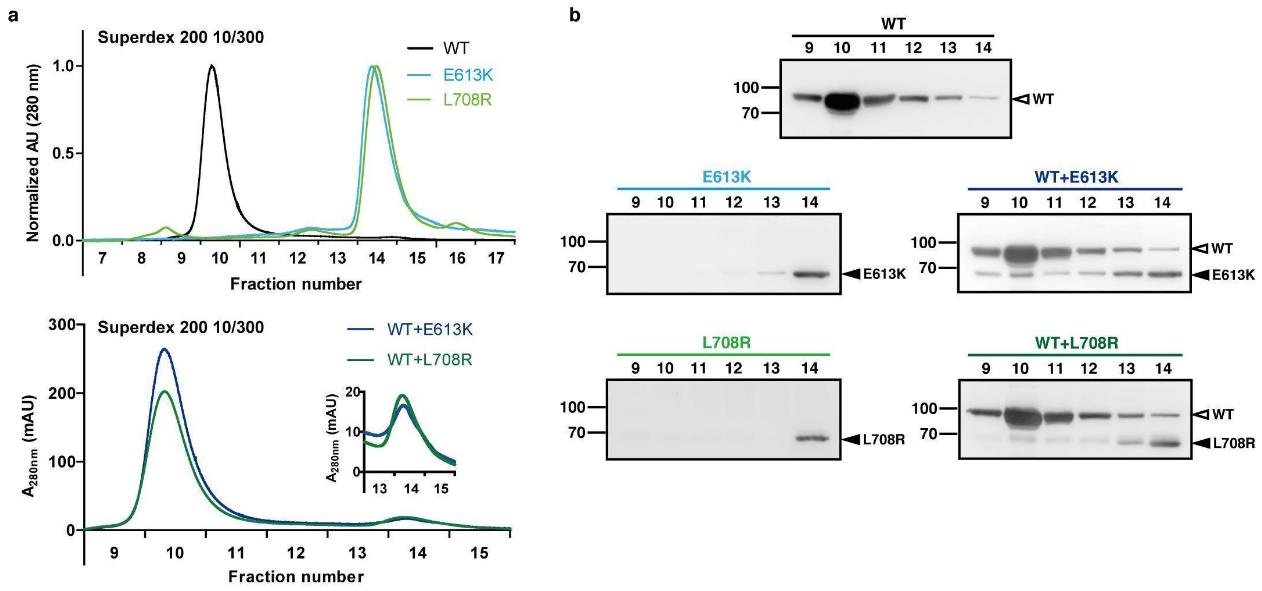

Supplementary Fig. 12: Binding of two designed monomeric MtaLon mutants to MtaLon-WT. a, The overlay chromatogram of size-exclusion chromatography of MtaLon-WT (WT, 20 μM), ΔN-E613K (E613K, 15 μM), and ΔN-L708R (L708R, 15 μM), which all carry a C-terminal 6xHis tag, is shown on the top. The overlay chromatogram of E613K and L708R incubated with WT is shown on the bottom. **b,** Western blot analysis of the fractions 9–14 from (**a**), detected with the 6xHis tag monoclonal antibody. Source data are provided as a Source Data file.

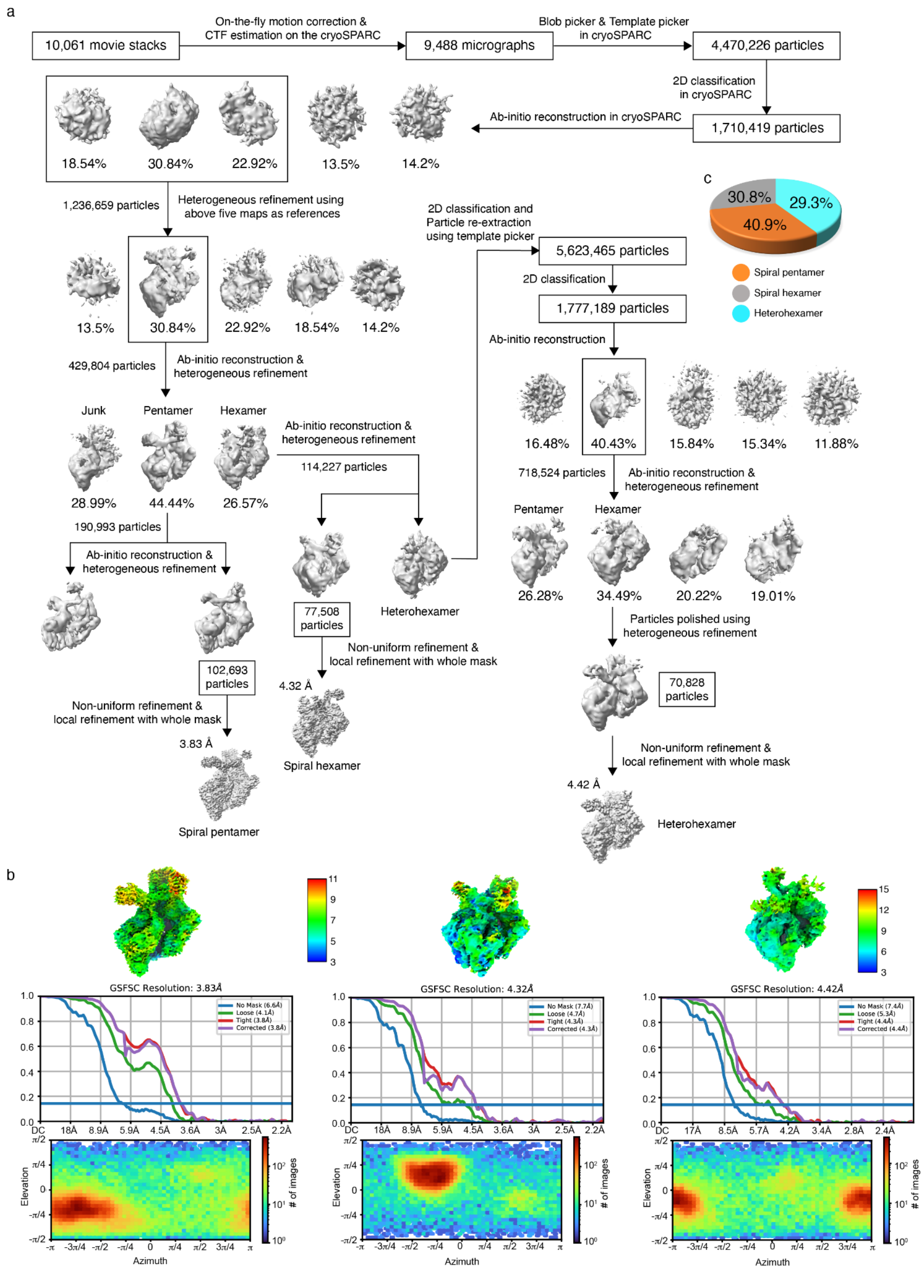

Supplementary Fig. 13: Single-particle cryo-EM analysis of MtaLon-Y224S: ΔN-E613K:ADP. a, Workflow of the data processing. **b,** Evaluation of the final 3D reconstructions. Top: Resolution maps for the final 3D reconstructions; middle: Gold standard FSC plots for the final 3D reconstructions, calculated in cryoSPARC; bottom: Euler angle distribution of the particle images. **c,** Pie chart showing the ratio of spiral penamers, spiral hexamers, and the heterohexamers (the *5+1* heterocomplex with ΔN-E613K).

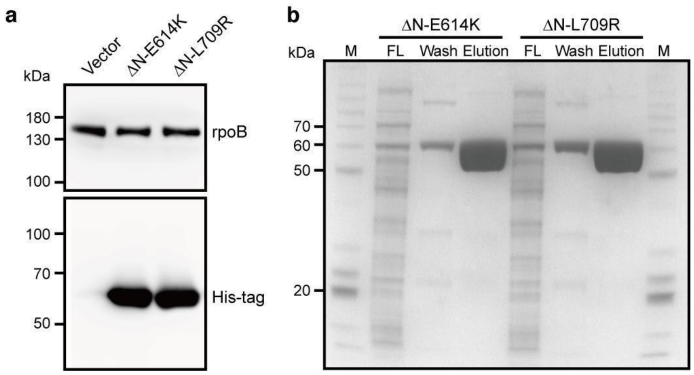

Supplementary Fig. 14: Protein expression of two monomeric mutants of EcoLon in *E. coli* MG1655 cells. a, Western-blot analysis of the cell lysates of MG1655 cells transformed with the vector, EcoLon-ΔN-E614K, or -ΔN-L709R plasmids and induced with 0.5% L-arabinose. Expressions of EcoLon-ΔN-E614K and -ΔN-L709R were detected with the 6xHis tag monoclonal antibody. RNA polymerase subunit beta (rpoB) was used for an internal loading control. **b,** Ni-chelation chromatography of recombinant 6xHis-tagged EcoLon-ΔN-E614K and -ΔN-L709R expressed in MG1655 cells assessed by Coomassie blue staining. M denotes the molecular weight marker. FL, Wash, and Elution represent samples taken from the flow-through, wash (with 25 mM imidazole), and elution fractions, respectively. Source data are provided as a Source Data file.

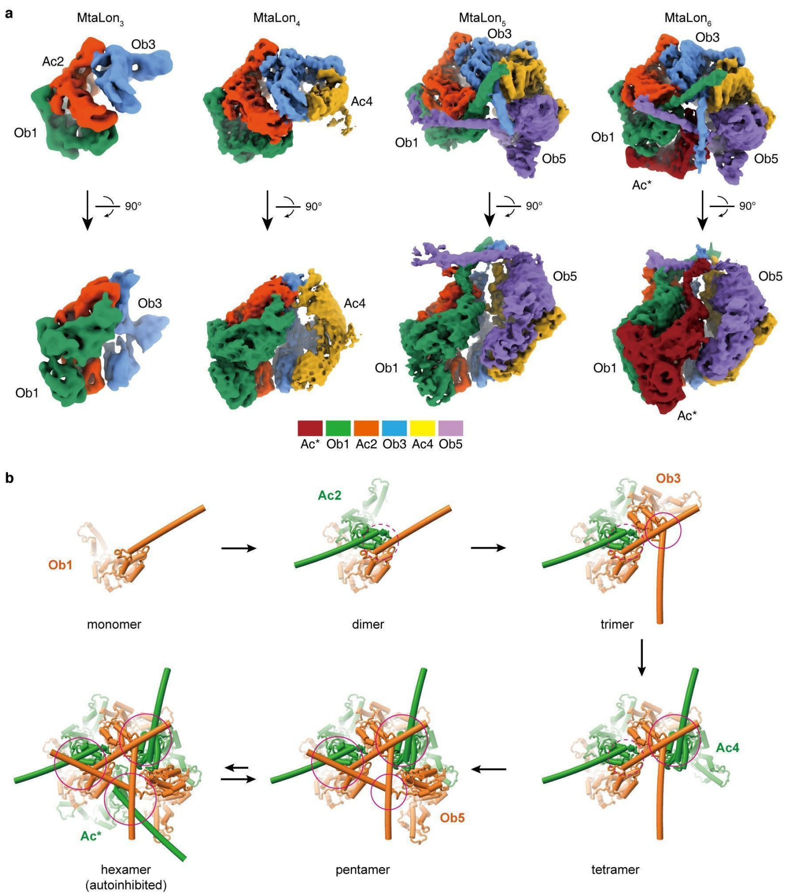

Supplementary Fig. 15: Assembly of the tensegrity helix triangle by clockwise sequential incorporation of protomers. a, Cryo-EM maps reconstructed from the MtaLon:ADP data reveal the trimeric, tetrameric, pentameric, and hexameric oligomers in left-handed spiral conformation. To highlight the tensegrity helix triangle (THT), the densities of the NGDs resolved in the maps of the pentamer and the hexamer are not shown in the displayed contour levels. **b,** The proposed assembly sequence is shown in the axial views. The NGDs are omitted for clarity. Dashed circles mark the antiparallel helical interactions involving two LHs; solid circles denote crossover helical interactions. The tensegrity helix triangle is constructed only upon the incorporation of the 5th protomer to form crossover helical interactions at the three vertices. The Ob-protomers and Ac-protomers are colored in orange and green, respectively.

Supplementary Table 1: Cryo-EM data collection, processing, and model validation of MtaLon-Y224S:ATPγS.

	**MtaLon-Y224S:ATPγS**	
**Data collection and processing**		
Microscope	FEI Titan Krios	
Voltage (kV)	300	
Camera	Gatan K3	
Grids Type	R1.2/1.3 Quantifoil copper grid (200 mesh)	
Sample concentration	0.5 mg/mL	
Magnification	105,000×	
C2 aperture size (μm)	50	
Objective aperture size (μm)	None	
Pixel size (Å)	0.83 (super-resolution: 0.415)	
Total exposure (e-/Å^2)	73.8	
Exposure time (s)	1.34	
Number of frames per exposure	50	
Energy filter slit width (eV)	18	
Data collection software	EPU 2.10	
Number of exposures per hole	2	
Defocus range (μm)	-1.2 to -2.0	
Number of micrographs collected	12,982	
Number of micrographs used	11,490	
Number of initial particles	4,721,627	
Conformations	**Pentamer**	**Hexamer**
Symmetry	C1	C1
Number of final particles	777,119	612,381
Resolution (0.143 gold standard FSC, Å)	3.7	3.8
Local resolution range (Å)	3 - 7	3 - 7
**Atomic model refinement**		
Software	phenix	phenix
Clashscore, all atoms	16.51	14.04
Poor rotamers (%)	0.15	0.13
Favored rotamers (%)	98.98	98.87
Ramachandran outliers (%)	0.05	0.02

Ramachandran favored (%)	91.52	93.6
MolProbity score	2.22	2.1
Bad bonds (%)	0	0
Bad angles (%)	0.08	0.12
CC box	0.81	0.80
**Accession numbers**		
EMDB	EMD-34000	EMD-34001
PDB	7YPH	7YPI

Supplementary Table 2: Cryo-EM data collection, processing, and model validation of MtaLon-Apo.

	**MtaLon-Apo**			
**Data collection and processing**				
Microscope	Titan Krios G3i			
Voltage (kV)	300			
Camera	Thermo Fisher Falcon 4			
Grids Type	R1.2/1.3 Quantifoil copper grid (200 mesh)			
Sample concentration	0.5 mg/mL			
Magnification	96,000×			
C2 aperture size (μm)	70			
Objective aperture size (μm)	100			
Pixel size (Å)	0.82			
Total exposure (e-/Å^2)	48			
Exposure time (s)	5.8			
Number of frames per exposure	40			
Energy filter slit width (eV)	15			
Data collection software	EPU 2.7			
Number of exposures per hole	6			
Defocus range (μm)	-1.0 to -2.5			
Number of micrographs collected	14,692			
Number of micrographs used	13,611			
Number of initial particles	1,361,155			
Conformations	**Trimer**	**Tetramer**	**Pentamer**	**Hexamer**
Symmetry	C1	C1	C1	C1
Number of final particles	156,915	109,756	285,591	122,515
Resolution (0.143 gold standard FSC, Å)	3.7	3.8	3.7	4.1
Local resolution range (Å)	3 - 7	3 - 7	3 - 7	3 - 7
**Atomic model refinement**				
Software	phenix	phenix	phenix	phenix
Clashscore, all atoms	6.61	8.84	10	11.68
Poor rotamers (%)	0.15	0.34	0.96	0.59
Favored rotamers (%)	96.01	96.05	94.58	95.76
Ramachandran outliers (%)	0.13	0.09	0.16	0.19

Ramachandran favored (%)	88.93	87.59	88.20	90.94
MolProbity score	1.94	2.08	2.12	2.10
Bad bonds (%)	0.01	0.01	0.03	0.01
Bad angles (%)	0.03	0.07	0.14	0.08
CC box	0.86	0.87	0.82	0.84
**Accession numbers**				
EMDB	EMD-34107	EMD-34108	EMD-34109	EMD-34110
PDB	7YUH	7YUM	7YUP	7YUT

Supplementary Table 3: Cryo-EM data collection, processing, and model validation of MtaLon:ADP.

	**MtaLon:ADP**			
**Data collection and processing**				
Microscope	Titan Krios G3i			
Voltage (kV)	300			
Camera	Gatan K3			
Grids Type	R1.2/1.3 Quantifoil copper grid (200 mesh)			
Sample concentration	0.5 mg/mL			
Magnification	105,000×			
C2 aperture size (μm)	70			
Objective aperture size (μm)	100			
Pixel size (Å)	0.82			
Total exposure (e-/Å^2)	60			
Exposure time (s)	1.5			
Number of frames per exposure	40			
Energy filter slit width (eV)	15			
Data collection software	EPU 2.7			
Number of exposures per hole	6			
Defocus range (μm)	-1.0 to -2.5			
Number of micrographs collected	6,969			
Number of micrographs used	6,858			
Number of initial particles	1,402,639			
Conformations	**Trimer**	**Tetramer**	**Pentamer**	**Hexamer**
Symmetry	C1	C1	C1	C1
Number of final particles	17,690	103,394	253,989	226,611
Resolution (0.143 gold standard FSC, Å)	5.8	3.8	3.6	3.8
Local resolution range (Å)	5 - 9	3 - 7	3 - 7	3 - 7
**Atomic model refinement**				
Software	phenix	phenix	phenix	phenix
Clashscore, all atoms	18.8	15.54	15.89	14.63
Poor rotamers (%)	0.15	0.06	0.15	0.26
Favored rotamers (%)	96.84	99.77	99.38	99.38
Ramachandran outliers (%)	0	0	0.05	0.09

Ramachandran favored (%)	91.01	93.73	91.73	93.58
MolProbity score	2.29	2.11	2.20	2.09
Bad bonds (%)	0	0	0	0
Bad angles (%)	0.04	0.01	0.03	0.02
CC box	0.89	0.78	0.81	0.82
**Accession numbers**				
EMDB	EMD-34111	EMD-34112	EMD-34113	EMD-34114
PDB	7YUU	7YUV	7YUW	7YUX

Supplementary Table 4: Cryo-EM data collection, processing, and model validation of MtaLon-S678A:casein:ADP.

	**MtaLon-S678A:casein:ADP**		
**Data collection and processing**			
Microscope	FEI Titan Krios		
Voltage (kV)	300		
Camera	Gatan K3		
Grids Type	R1.2/1.3 Quantifoil copper grid (200 mesh)		
Sample concentration	0.5 mg/mL		
Magnification	81,000×		
C2 aperture size (µm)	50		
Objective aperture size (µm)	None		
Pixel size (Å)	1.061 (super-resolution: 0.5305)		
Total exposure (e-/Å^2)	59		
Exposure time (s)	2.52		
Number of frames per exposure	60		
Energy filter slit width (eV)	20		
Data collection software	EPU 2.10		
Number of exposures per hole	2		
Defocus range (µm)	-1.4 to -2.2		
Number of micrographs collected	12,546		
Number of micrographs used	11,716		
Number of initial particles	1,756,270		
Conformations	**Pentamer**	**Hexamer**	**Close-hexamer**
Symmetry	C1	C1	C1
Number of final particles	486,028	100,882	207,760
Resolution (0.143 gold standard FSC, Å)	3.8	4.7	3.4
Local resolution range (Å)	4 - 8	4 - 8	3 - 7
**Atomic model refinement**			
Software	phenix		phenix
Clashscore, all atoms	19		20.29
Poor rotamers (%)	0.12		0.46
Favored rotamers (%)	98.73		97.54
Ramachandran outliers (%)	0.08		0.06

Ramachandran favored (%)	91.47		95.3
MolProbity score	2.28		2.13
Bad bonds (%)	0		0.03
Bad angles (%)	0.1		0.04
CC box	0.87		0.80
**Accession numbers**			
EMDB	EMD-34002	EMD-34004	EMD-34003
PDB	7YPJ	N/A	7YPK

Supplementary Table 5: Cryo-EM data collection, processing, and model validation of MtaLon-Y397A/S678A:casein:ATPγS.

	**MtaLon-Y397A/S678A:casein:ATPγS**	
**Data collection and processing**		
Microscope	FEI Titan Krios	
Voltage (kV)	300	
Camera	Gatan K3	
Grids Type	R1.2/1.3 Quantifoil copper grid (200 mesh)	
Sample concentration	0.5 mg/mL	
Magnification	81,000×	
C2 aperture size (μm)	50	
Objective aperture size (μm)	None	
Pixel size (Å)	1.061 (super-resolution: 0.5305)	
Total exposure (e-/Å^2)	48	
Exposure time (s)	2	
Number of frames per exposure	50	
Energy filter slit width (eV)	18	
Data collection software	EPU 2.10	
Number of exposures per hole	2	
Defocus range (μm)	-1.4 to -2.2	
Number of micrographs collected	5,554	
Number of micrographs used	4,941	
Number of initial particles	1,423,777	
Conformations	**Pentamer**	**Hexamer**
Symmetry	C1	C1
Number of final particles	218,990	144,561
Resolution (0.143 gold standard FSC, Å)	3.72	4.08
Local resolution range (Å)	3 - 7	3 - 7
**Accession numbers**		
EMDB	EMD-34005	EMD-34006

Supplementary Table 6: Cryo-EM data collection, processing, and model validation of MtaLon-M217A:casein:ADP.

	**MtaLon-M217:casein:ADP**	
**Data collection and processing**		
Microscope	FEI Titan Krios	
Voltage (kV)	300	
Camera	Gatan K3	
Grids Type	R1.2/1.3 Quantifoil copper grid (200 mesh)	
Sample concentration	1 mg/mL	
Magnification	81,000×	
C2 aperture size (μm)	50	
Objective aperture size (μm)	None	
Pixel size (Å)	1.061 (super-resolution: 0.5305)	
Total exposure (e-/Å^2)	49	
Exposure time (s)	2	
Number of frames per exposure	50	
Energy filter slit width (eV)	18	
Data collection software	EPU 2.10	
Number of exposures per hole	2	
Defocus range (μm)	-1.4 to -2.2	
Number of micrographs collected	10,600	
Number of micrographs used	10,361	
Number of initial particles	1,123,198	
Conformations	**Pentamer**	**Hexamer**
Symmetry	C1	C1
Number of final particles	74,613	60,769
Resolution (0.143 gold standard FSC, Å)	4.3	5.3
Local resolution range (Å)	5 - 9	5 - 9
**Accession numbers**		
EMDB	EMD-34116	EMD-34117

Supplementary Table 7: Cryo-EM data collection, processing, and model validation of MtaLon-Y224S:ΔN-E613K:ADP.

	**MtaLon-Y224S:ΔN-E613K:ADP**		
**Data collection and processing**			
Microscope	FEI Titan Krios		
Voltage (kV)	300		
Camera	Gatan K3		
Grids Type	R1.2/1.3 Quantifoil copper grid (200 mesh)		
Sample concentration	0.5 mg/mL		
Magnification	81,000×		
C2 aperture size (μm)	50		
Objective aperture size (μm)	None		
Pixel size (Å)	1.061 (super-resolution: 0.5305)		
Total exposure (e-/Å^2)	52		
Exposure time (s)	1.8		
Number of frames per exposure	50		
Energy filter slit width (eV)	10		
Data collection software	EPU 3.3.1.5184REL		
Number of exposures per hole	2		
Defocus range (μm)	-1.4 to -2.2		
Number of micrographs collected	10,061		
Number of micrographs used	9,488		
Number of initial particles	1,710,419		
Conformations	**Pentamer**	**Hexamer**	***5+1*** **heterocomplex**
Symmetry	C1	C1	C1
Number of final particles	102,693	77,508	70,828
Resolution (0.143 gold standard FSC, Å)	3.8	4.3	4.4
Local resolution range (Å)	3 - 11	3 - 11	4 - 15
**Atomic model refinement**			
Software			phenix
Clashscore, all atoms			17.66
Poor rotamers (%)			0.05
Favored rotamers (%)			98.83
Ramachandran outliers (%)			0.02

Ramachandran favored (%)			93.09
MolProbity score			2.19
Bad bonds (%)			0
Bad angles (%)			0.06
CC box			0.86
**Accession numbers**			
EMDB	EMD-36865	EMD-36866	EMD-36867
PDB	N/A	N/A	8K3Y
