# Supplementary material for: A 5+1 assemble-to-activate mechanism of the Lon proteolytic machine
Source: Nat Commun. 2023 Nov 13;14:7340. doi: 10.1038/s41467-023-43035-2 (PMC10643698; doi:10.1038/s41467-023-43035-2)
Supplement: Supplementary file 3 — Reporting Summary [file 41467_2023_43035_MOESM3_ESM.pdf]

nature portfolio

Corresponding author(s): Chung-I Chang

Last updated by author(s): Oct 11, 2023

Reporting Summary

Nature Portfolio wishes to improve the reproducibility of the work that we publish. This form provides structure for consistency and transparency in reporting. For further information on Nature Portfolio policies, see our Editorial Policies and the Editorial Policy Checklist.

Statistics

For all statistical analyses, confirm that the following items are present in the figure legend, table legend, main text, or Methods section.

n/a	Confirmed	
☐	☒	The exact sample size (*n*) for each experimental group/condition, given as a discrete number and unit of measurement
☐	☒	A statement on whether measurements were taken from distinct samples or whether the same sample was measured repeatedly
☐	☒	The statistical test(s) used AND whether they are one- or two-sided<br>*Only common tests should be described solely by name; describe more complex techniques in the Methods section.*
☒	☐	A description of all covariates tested
☐	☒	A description of any assumptions or corrections, such as tests of normality and adjustment for multiple comparisons
☐	☒	A full description of the statistical parameters including central tendency (e.g. means) or other basic estimates (e.g. regression coefficient) AND variation (e.g. standard deviation) or associated estimates of uncertainty (e.g. confidence intervals)
☐	☒	For null hypothesis testing, the test statistic (e.g. *F*, *t*, *r*) with confidence intervals, effect sizes, degrees of freedom and *P* value noted<br>*Give P values as exact values whenever suitable.*
☒	☐	For Bayesian analysis, information on the choice of priors and Markov chain Monte Carlo settings
☒	☐	For hierarchical and complex designs, identification of the appropriate level for tests and full reporting of outcomes
☒	☐	Estimates of effect sizes (e.g. Cohen's *d*, Pearson's *r*), indicating how they were calculated

Our web collection on statistics for biologists contains articles on many of the points above.

Software and code

Policy information about availability of computer code

Data collection	I-control'" microplate reader software was used for detecting fluorescence polarization. EPU 3.3.I.5184REL, EPU 2.7, and EPU 2.10 were used for cryo-EM data collection. LAS-4000mini imaging system was used for acquiring western blot and SDS-PAGE gel images. XL-A analytical ultracentrifuge was used for SV-AUC. mini DAWN TREOS detector, Agilent 1260 Infinity HPLC, and Optilab T-rEX differential refractive index detector were used for SEC-MALs. Olympus scanR high-content screen station was used for filamentation imaging.
Data analysis	MotionCor2, Relion 3.1, CTFFIND4, and cryoSPARC 3.2 were used for 3D reconstruction of cryo-EM data. ImageJ with the plug-in MicrobeJ was used for cell size measurement. ASTRA 6 software was used for SEC-MALs analysis. Sedfit 16.lc and SEDNTERP were used for SV-AUC analysis. SWISS-MODEL server, MDFF, ChimeraX 1.2.5, coot 0.9.6, phenix.real_space_refine (phenix 1.18.2), LigandFit (phenix 1.18.2), phenix.map_model_cc (phenix 1.18.2), and MolProbity 4.5.1 were used for model building and validation. Graph Pad Prism 9.1.2 was used for statistical analysis.

For manuscripts utilizing custom algorithms or software that are central to the research but not yet described in published literature, software must be made available to editors and reviewers. We strongly encourage code deposition in a community repository (e.g. GitHub). See the Nature Portfolio guidelines for submitting code & software for further information.

Data

Policy information about availability of data

All manuscripts must include a data availability statement. This statement should provide the following information, where applicable:

- Accession codes, unique identifiers, or web links for publicly available datasets
- A description of any restrictions on data availability
- For clinical datasets or third party data, please ensure that the statement adheres to our policy

Source data are provided with this paper. The accession numbers of the cryo-EM maps of MtaLon-Y224S:ATPγS, deposited in the Electron Microscopy Data Bank (EMDB), for the spiral pentamer and spiral hexamer are EMD-34000 and EMD-34001, respectively; the coordinates of the atomic models for the spiral pentamer and spiral hexamer are deposited in the Protein Data Bank (PDB) with the PDB codes 7YPH and 7YPI, respectively. The accession numbers of the cryo-EM maps of MtaLon-Apo for the spiral oligomers of trimer, tetramer, pentamer, and hexamer are EMD-34107, EMD-34108, EMD-34109, and EMD-34110, respectively; the PDB codes for the spiral oligomers of trimer, tetramer, pentamer, and hexamer are 7YUH, 7YUM, 7YUP, and 7YUT, respectively. The accession numbers of the cryo-EM maps of MtaLon:ADP for the spiral oligomers of trimer, tetramer, pentamer, and hexamer are EMD-34111, EMD-34112, EMD-34113, and EMD-34114, respectively; the PDB codes for the spiral oligomers of trimer, tetramer, pentamer, and hexamer are 7YUU, 7YUV, 7YUW, and 7YUX, respectively. The accession numbers of the cryo-EM maps of MtaLon-S678A:casein:ADP for the spiral pentamer, spiral hexamer, and close-ring hexamer are EMD-34002, EMD-34004, and EMD-34003, respectively; the PDB codes for the spiral pentamer and close-ring hexamer are 7YPJ and 7YPK, respectively. The accession numbers of the cryo-EM maps of MtaLon-Y397A/S678A:casein:ATPγS for the spiral pentamer and spiral hexamer are EMD-34005 and EMD-34006, respectively. The accession numbers of the cryo-EM maps of MtaLon-M217A:casein:ADP for the spiral pentamer and spiral hexamer are EMD-34116 and EMD-34117, respectively. The accession numbers of the cryo-EM maps of MtaLon-Y224S:ΔN-E613K:ADP for the spiral pentamer, spiral hexamer, and the 5+1 heterocomplex are EMD-36865, EMD-36866, and EMD-36867, respectively; the PDB code for the 5+1 heterocomplex is 8K3Y.

Research involving human participants, their data, or biological material

Policy information about studies with human participants or human data. See also policy information about sex, gender (identity/presentation), and sexual orientation and race, ethnicity and racism.

Reporting on sex and gender	N/A
Reporting on race, ethnicity, or other socially relevant groupings	N/A
Population characteristics	N/A
Recruitment	N/A
Ethics oversight	N/A

Note that full information on the approval of the study protocol must also be provided in the manuscript.

Field-specific reporting

Please select the one below that is the best fit for your research. If you are not sure, read the appropriate sections before making your selection.

[x] Life sciences [ ] Behavioural & social sciences [ ] Ecological, evolutionary & environmental sciences

For a reference copy of the document with all sections, see nature.com/documents/nr-reporting-summary-flat.pdf

Life sciences study design

All studies must disclose on these points even when the disclosure is negative.

Sample size	Sample sizes were chosen based on similar experiments reported in the literature.
Data exclusions	No data were excluded.
Replication	Cryo-EM reconstruction of a protein specimen was not replicated because the 3D map derived was validated successfully later by other experiments. All attempts at replication in the other experiments were successful.
Randomization	This is not relevant to our study, which involves determination of 3D protein structures and validating the structural models by mutational analysis.
Blinding	This is not relevant to our study, which involves determination of 3D protein structures and validating the structural models by mutational analysis.

Reporting for specific materials, systems and methods

We require information from authors about some types of materials, experimental systems and methods used in many studies. Here, indicate whether each material, system or method listed is relevant to your study. If you are not sure if a list item applies to your research, read the appropriate section before selecting a response.

Materials & experimental systems

n/a	Involved in the study	
☐	☒	Antibodies
☒	☐	Eukaryotic cell lines
☒	☐	Palaeontology and archaeology
☒	☐	Animals and other organisms
☒	☐	Clinical data
☒	☐	Dual use research of concern
☒	☐	Plants

Methods

n/a	Involved in the study	
☒	☐	ChIP-seq
☒	☐	Flow cytometry
☒	☐	MRI-based neuroimaging

Antibodies

Antibodies used	Primary Ab:<br>6x-His tag monoclonal antibody (clone HIS.H8, Abcam #ab18184, 1:10000); anti-E. coli RNA polymerase beta antibody (clone 8RB13, Biolegend #663903, 1:10000).<br>Secondary Ab:<br>HRP-conjugated anti-mouse antibody (Abcam #ab6789, 1:20000).
Validation	1. 6x-His tag monoclonal antibody: https://www.abcam.com/products/primary-antibodies/6x-his-tag-antibody-hish8-ab18184.html<br>2. HRP-conjugated anti-mouse antibody: https://www.abcam.com/products/secondary-antibodies/goat-mouse-igg-hl-hrp-ab6789.html<br>3. anti-E. coli RNA polymerase beta antibody : https://www.biolegend.com/en-gb/products/anti-e-coli-rna-polymerase-beta-antibody-10494?GroupID=GROUP26

Plants

Seed stocks	N/A
Novel plant genotypes	N/A
Authentication	N/A
